# Critical Electrochemistry Technologies Applicable in Space Exploration

**DOI:** 10.1002/advs.202504447

**Published:** 2025-06-23

**Authors:** Ruisi Xu, Zepeng Lv, Shaolong Li, Jilin He, Jianxun Song

**Affiliations:** ^1^ Zhongyuan Critical Metals Laboratory Zhengzhou University Zhengzhou Henan 450001 P. R. China; ^2^ School of Material Science and Engineering Zhengzhou University Zhengzhou Henan 450001 P. R. China; ^3^ National Key Laboratory of Special Rare Metal Materials Zhengzhou University Zhengzhou Henan 450001 P. R. China; ^4^ State Key Laboratory of Critical Metals Beneficiation, Metallurgy and Purification Zhengzhou University Zhengzhou Henan 450001 P. R. China

**Keywords:** electrochemistry, energy storage, extraterrestrial metallurgy, resource recycling, space exploration, space resource

## Abstract

Electrochemistry is versatile, less subject to external influences, and has great potential for the exploration of the unpredictable space environment. This work analyzes the available resources in space and discusses the application scenarios and challenges of electrochemical methods in four areas: metallurgical processing of mineral resources, water resources utilization, the carbon cycle in daily extraterrestrial life, and electrical energy storage. By examining these pivotal areas, this work elucidates the unique advantages and limitations of electrochemical technologies in space exploration. Furthermore, the paper explores the innovative application scenarios of electrochemistry in space, presenting fresh insights into how these technologies can be adapted and optimized for extraterrestrial environments. This comprehensive analysis not only provides a strategic direction for the breakthrough and advancement of electrochemical applications in space but also serves as an invaluable reference for the development of human extraterrestrial exploration and resource harvesting programs, guiding future research endeavors and technological innovations in this fascinating frontier.

## Graphical abstract

The available resources in space and discuss the application scenarios and challenges of electrochemical methods in four critical domains is analyzed: metallurgical processing of mineral resources, water resources utilization, carbon cycle management in extraterrestrial habitats, and electrical energy storage. By examining these pivotal areas, the unique advantages and inherent limitations of electrochemical approaches in space applications is highlighted.

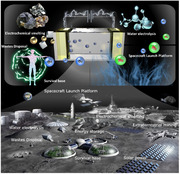

## 1 Introduction

Human fascination with space dates back to ancient times, with astronomical observations playing a key role in the evolution of early civilizations. The advent of modern astronomy began in 1610 when Galileo used a telescope to observe Jupiter's moons, marking a new era of scientific exploration.^[^ ^1^ ^]^ In the early 20th century, Konstantin Tsiolkovsky laid the theoretical groundwork for astronautics, envisioning space stations, artificial gravity, and interplanetary travel.^[^ ^2^ ^]^ Subsequent developments, including Robert Goddard's concept of multi‐stage rockets, propelled spaceflight from theory to reality.^[^ ^3^ ^]^

The realization of the first liquid‐fueled rocket in 1926 and the historic lunar landing by American astronauts in 1969 marked significant milestones in human space exploration.^[^ ^4^ ^]^ The analysis of the lunar soil brought back by several missions to the moon after the modern era, complemented by the results of advanced telescopes and remote sensing satellites, has revealed that the main composition of the lunar surface is basalt rich in Fe, Mg, and Ti. Remarkably, in some lunar regions, the TiO_2_ content of basalts is higher than 4.5%, up to ≈70–100 trillion tonnes. Additionally, the amount of titanium in the surface is generally higher than that of the Earth, along with significant quantities of Al (10–18 wt.%), a large amount of Si, and a concentration of U, Lu, Eu, etc., of which the fusion fuel ^3^He can reach 100–500 tons.^[^ ^5^ ^]^

Mars, as the closest Earth‐like planet, has been a target of successful landings since the former Soviet Union's “Mars 1” probe in 1960, culminating in China's Tianwen 1 probe landing on Mars in 2021. Researching on the shape of the surface of Mars and its composition is constantly advancing. To the best of our knowledge, the surface of Mars is also covered with large amounts of iron oxide ores^[^ ^6^ ^]^ and water ice.^[^ ^7^ ^]^ In addition, Near‐Earth Objects (NEOs) also contain large amounts of mineral resources such as S, SiO_2_, CaO, and various metals, etc. The mineral content of these NEOs is even hundreds of times higher than that of the lunar or Martian soil, and contains elements that are scarce on the Moon or Mars.^[^ ^8^ ^]^

The abundance of these space resources stand in stark contrast to the increasingly scarce resources on Earth, and they offer the potential for directly utilization in space mining sites for long‐term space exploration or collected and transferred back to Earth for further use. For this reason, various nations and organizations have formulated a series of strategies on the exploration and utilization of space resources. In 2015, the United States formulated the “Space Resource Exploration and Utilization Act of 2015”, and the European Space Agency (ESA) announced the construction plans for a “lunar village” in 2016. More recently, in 2021, China and Russia jointly released the “Roadmap of International Lunar Research Station V1.0” and “Guide to International Lunar Research Station Partner V1.0”,^[^ ^9^ ^]^ marking a significant step toward international cooperation in lunar exploration.

**Figure 1:**
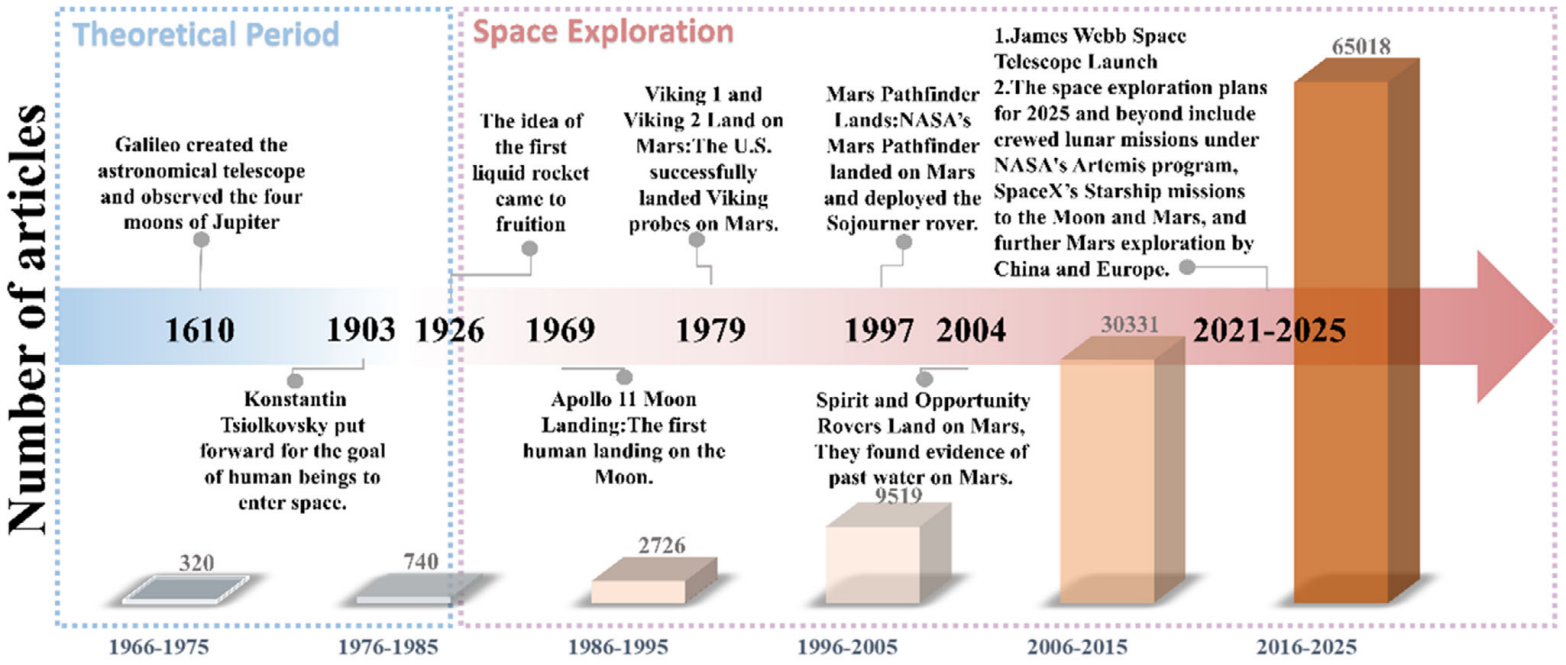
The historical process of human space exploration and number of articles published related to space exploration, spanning from 1975–2025 (Credit: ISI Web of Knowledge).

Furthermore, the volume of academic research on space exploration has experienced exponential growth (**Figure** **1**), demonstrating the increasing global interest and scientific commitment to advancing this field. Looking ahead, the focus on space resource utilization is expected to accelerate, with plans including crewed lunar missions under NASA's Artemis program, SpaceX's Starship missions targeting the Moon and Mars, and expanded Martian exploration initiatives by China and Europe. These efforts, as summarized in Figure 1, not only represent critical milestones in the history of human space exploration but also lay a robust foundation for future generations to sustainably harness the immense potential of space resources.

Deep space exploration is the next “great age of navigation” for humanity. As the heat, intensity, scope, and depth of deep space exploration activities continue to increase, the traditional means of transportation will be difficult to support the development of future space missions. Consequently, there is a pressing need to continuously reduce the dependence on Earth's material and energy supplies. According to estimations, the cost of transporting resources from the Earth to the Moon is 100 K$/Ib, while the cost of traveling to Mars is 500 K$/Ib.^[^ ^10^ ^]^ Therefore, the development of *in‐situ* resource utilization (ISRU) technology is imperative. ISRU technology mainly includes the extraction, transformation, storage, and utilization of multiple resources under specific extraterrestrial environments, which runs through the whole process of space activities. Especially, it has been listed as the first technology prioritized for manned deep space exploration by the National Aeronautics and Space Administration (NASA) of the United States. Similarly, it is also designated as one of the six technological priority areas for development by China's manned deep space exploration team.

Among the various technological approaches to ISRU, electrochemical engineering has emerged as a particularly versatile and scalable solution. Its applications span high‐efficiency metal extraction (e.g., Ti, Al, Fe) via molten salt electrolysis, oxygen generation through electrochemical reduction of regolith, and the in‐situ fabrication of functional coatings and membranes. Unlike thermochemical or mechanical methods, electrochemical processes exhibit unique advantages under low‐gravity, vacuum, and radiation‐rich conditions: they are modular, gravity‐insensitive, and readily powered by solar energy—a critical asset in extraterrestrial environments. Furthermore, the maturing ecosystem of electrochemical energy technologies on Earth, including electrolyzers, fuel cells, and electrochemical reactors, provides a robust technological foundation for adaptation to off‐world systems.^[^ ^11^, ^12^, ^13^, ^14^, ^15^, ^16^, ^17^, ^18^, ^19^ ^]^

In light of these advantages, this review aims to systematically explore the principles, progress, and prospects of electrochemical ISRU (E‐ISRU) for space applications. Emphasis is placed on recent advancements in molten salt electro‐metallurgy, water electrolysis, CO_2_ conversion, and electrochemical energy storage and conversion technologies. By synthesizing insights from both planetary science and electrochemical engineering, this work offers a comprehensive framework to support the development of autonomous, self‐sustaining energy and material systems for long‐term space exploration.

## 2 The Utilization of Extraterrestrial Mineral Resource

**Figure 2:**
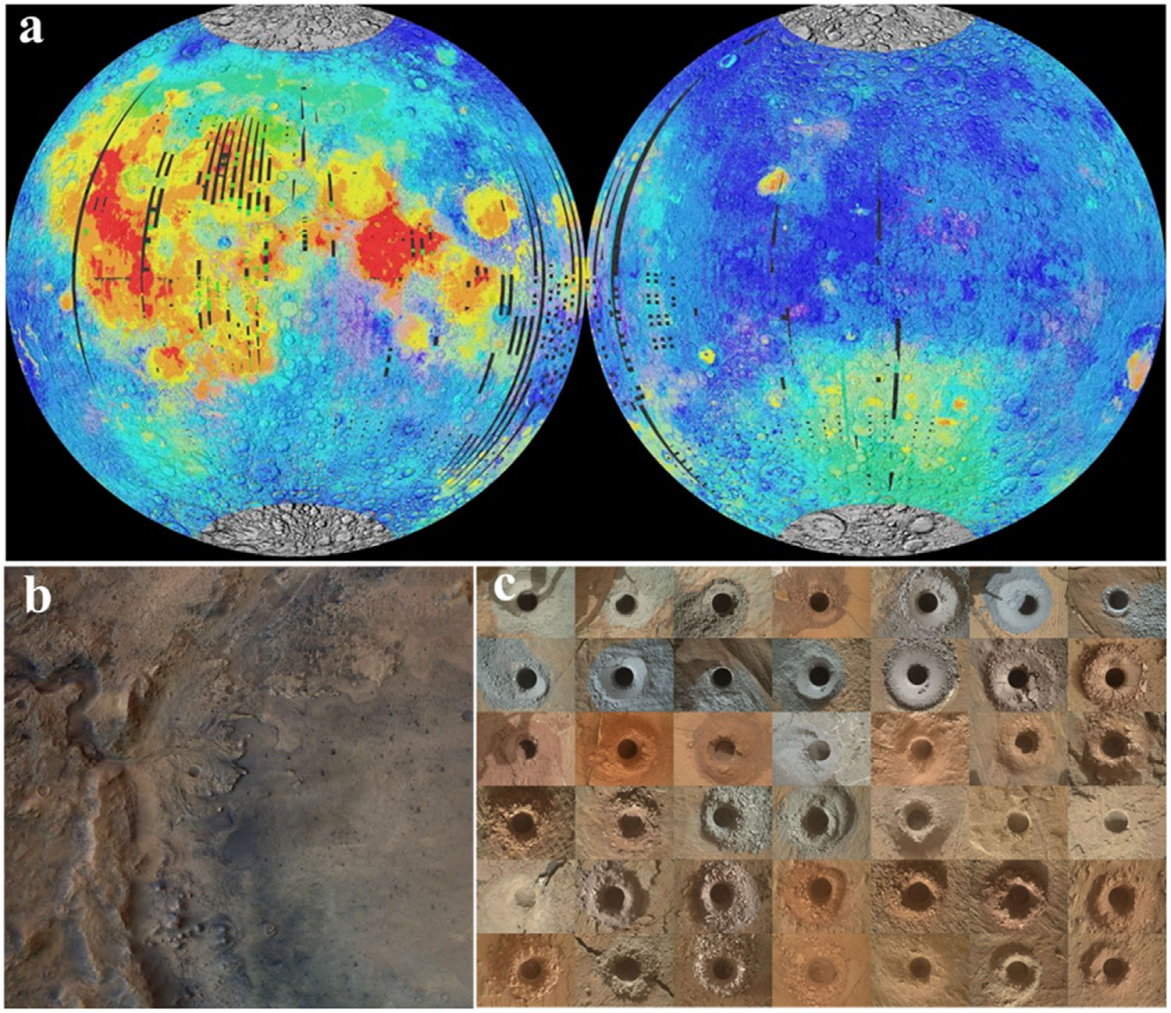
a) Distribution of rock types on the near (left) and far (right) sides of the Moon. Blue: plagioclase highlands; yellow: low‐titanium basalt; red: high‐titanium basalt. The large yellow/green area in the far southern hemisphere is the Antarctic‐Aitken basin. Image courtesy of Paul Spudis. Data from refs.[47, 48] b) A well‐preserved fluvial delta within Mars’ Jezero Crater. Image courtesy of ESA/DLR/FU‐Berlin.^[^ ^28^ ^]^ c) Grid displaying the 42 drill holes created by NASA's Curiosity rover on Mars, each corresponding to a distinct rock sampling site. Image courtesy of NASA/JPL‐Caltech/MSSS.

As society, productivity, and technology evolve, the demand for metals continues to grow. From 1950 to 2012, the global demand for metals increased at an unprecedented rate during the sixth decades of the century, and projections indicate that the global demand for metal resources will double between 2010 and 2030.^[^ ^20^, ^21^, ^22^, ^23^ ^]^ Most kind of mineral resources, as non‐renewable resources, need thousands of years to form through geological action. Similarly, the unique metal characteristics make them difficult to replace. Hence, the exploration of additional resources and the in‐situ utilization of the minerals have become the world's concern. In the exploration of the universe, different planets have their own unique mineral resources due to their distance from the fixed stars. For instance, samples of lunar soil and rocks in the near‐equatorial region obtained from Apollo's multiple lunar exploration missions, as well as the latest analysis of the samples collected by Chang'e 5 in the east‐northeast direction of Lümcke Mountain, show that the collected basalt primarily consist of monoclinic pyroxene (a kind of ionic material with a primary composition of XY(Si, Al)_2_O_6_, where X stands for calcium, sodium, magnesium, and Fe^2+^, and zinc, manganese, lithium species, and so on, while Y represents smaller ions such as chlorine, aluminum, Fe^3+^, vanadium, scandium, and others), plagioclase feldspar (with a major composition of Na[AlSi_3_O_8_]‐Ca[Al_2_Si_2_O_8_]), olivine (with a major composition of (Mg, Fe)_2_[SiO_4_]), and titanomagnetite (with a major composition of FeTiO_3_), along with minor quantities of quartz and cubic quartz (**Figure** **2**a).^[^ ^24^, ^25^, ^26^ ^]^

Mars, Earth's neighboring planet, has been extensively investigated through high‐resolution orbital imaging, infrared spectroscopy, and in‐situ surface analyses. These efforts have revealed a variety of geomorphological and mineralogical features shaped by prolonged aqueous activity and volcanism. For instance, a well‐preserved fluvial delta in Jezero Crater shows sedimentary stratification attributed to sustained water flow and deposition processes (Figure 2b), while a systematic compilation of over 40 drill holes in Gale Crater has uncovered diverse lithologies ranging in texture, color, and geochemical composition (Figure 2c).^[^ ^27^, ^28^ ^]^ These studies have confirmed the widespread presence of hematite, phyllosilicates, olivine, sulfates, and other secondary minerals that are indicative of past hydrothermal activity and geochemical weathering.^[^ ^29^, ^30^ ^]^

Utilizing these extraterrestrial materials for in‐situ resource production near space stations is potentially far more cost‐effective than mining and transporting Earth's limited resources. Elements such as magnesium, aluminum, iron, and titanium can be extracted directly from Lunar or Martian soils and rocks. These metals, as well as their alloys, have a high potential for use in construction materials, radiation barriers, and machine parts. For example, magnesium and its alloys have excellent properties, including low thermal conductivity (160 W/m·K) and magnetization (6.9 × 10^−9^ m^3^ Kg^−1^). It has a certain degree of electromagnetism and radiation shielding, with high damping capabilities, could be used as a barrier on the outside of the substrate in space, withstanding meteorite bombardment or absorbing seismic energy. Alternatively, they can be employed in the magnesium‐based battery with a high energy density at a low weight. A blend of magnesium chloride and magnesium oxide, known as magnesium chloride‐oxygen cement (SOREL cement), exhibits a compressive strength of 75 MPa, fast hardening, good adhesion, and abrasion‐resistant, making it suitable for the construction of extraterrestrial buildings.^[^ ^31^, ^32^, ^33^, ^34^ ^]^ Aluminum alloys are renowned for their high strength at low temperatures, high ductility, and resistance to sublimation at high temperatures or in vacuum conditions, playing an important role as early as the Apollo project.^[^ ^35^, ^36^, ^37^ ^]^ Titanium alloys can be used in environments where magnesium, aluminum, and steel are unsuitable, such as at high temperatures or under high workloads.^[^ ^38^ ^]^ Other metals and their alloys can also be used in space construction. For example, Szilard has proposed the use of beryllium alloys in the design of prefabricated spherical habitats, which have an average yield strength of 220 MPa and excellent resistance to radiation exposure as well as temperature.^[^ ^39^ ^]^

Moreover, many types of rocks, particularly silicate minerals, are rich in silicon. As a crucial material, silicon finds extensive applications in semiconductor technology, fiber‐reinforced construction materials, and as a fundamental component in solar energy conversion. Typically, the processing of silicon and silicon alloys is more efficient in the molten state, while the introduction of electrochemical techniques can significantly enhance energy utilization efficiency, improve product purity, and enable precise control over material morphology.^[^ ^40^, ^41^, ^42^ ^]^ Furthermore, in the microgravity environment of space, the reduction of gravitational forces may suppress natural convection, potentially influencing the deposition behavior of silicon; however, the exact mechanisms require further investigation. During molten salt electrolysis, dissolved Si^4+^ ions in the electrolyte migrate to the cathode, where they gain electrons and are reduced to form high‐purity elemental silicon.^[^ ^43^ ^]^ Simultaneously, oxygen gas released at the anode can be utilized in life support systems for space missions, while the residual molten silicate byproducts can serve as auxiliary materials for space construction, such as in additive manufacturing (e.g., 3D‐printed structural components) or ceramic composite materials.^[^ ^44^, ^45^ ^]^

Electrochemical metallurgy is the smelting technology using electrode reactions. The electrolyte (such as an aqueous solution or molten salt) will connect the cathode (represented by sulfide and oxide minerals) and anode (often an inert electrode) to drive redox reactions, thereby facilitating the metallurgical process of metal extraction. According to the temperature applied in the electrochemical reaction, the electrolytes used in the electrochemical extraction process of metals can be divided into three types: room‐temperature electrolytes (application temperature ≤100 °C, e.g. aqueous solutions, ionic liquids, etc.), high‐temperature molten salts (100 °C ≤ application temperature ≤1000 °C, such as alkali or alkaline‐earth metal chloride, fluoride, hydroxide or carbonate, etc.), ultra‐high‐temperature melts (1000 °C ≤ application temperature, such as molten oxides or sulfides).^[^ ^19^, ^46^ ^]^ In the case of aqueous solutions, ionic liquids, etc., the presence of hydrogen ions and hydroxyl radicals does not provide a wide enough electrochemical window for direct electrolysis of metals more active than hydrogen. Therefore, only high‐temperature and ultra‐high‐temperature melts are described below for space mineral resource smelting applications.

### 2.1 High Temperature Molten Salt Method

**Figure 3:**
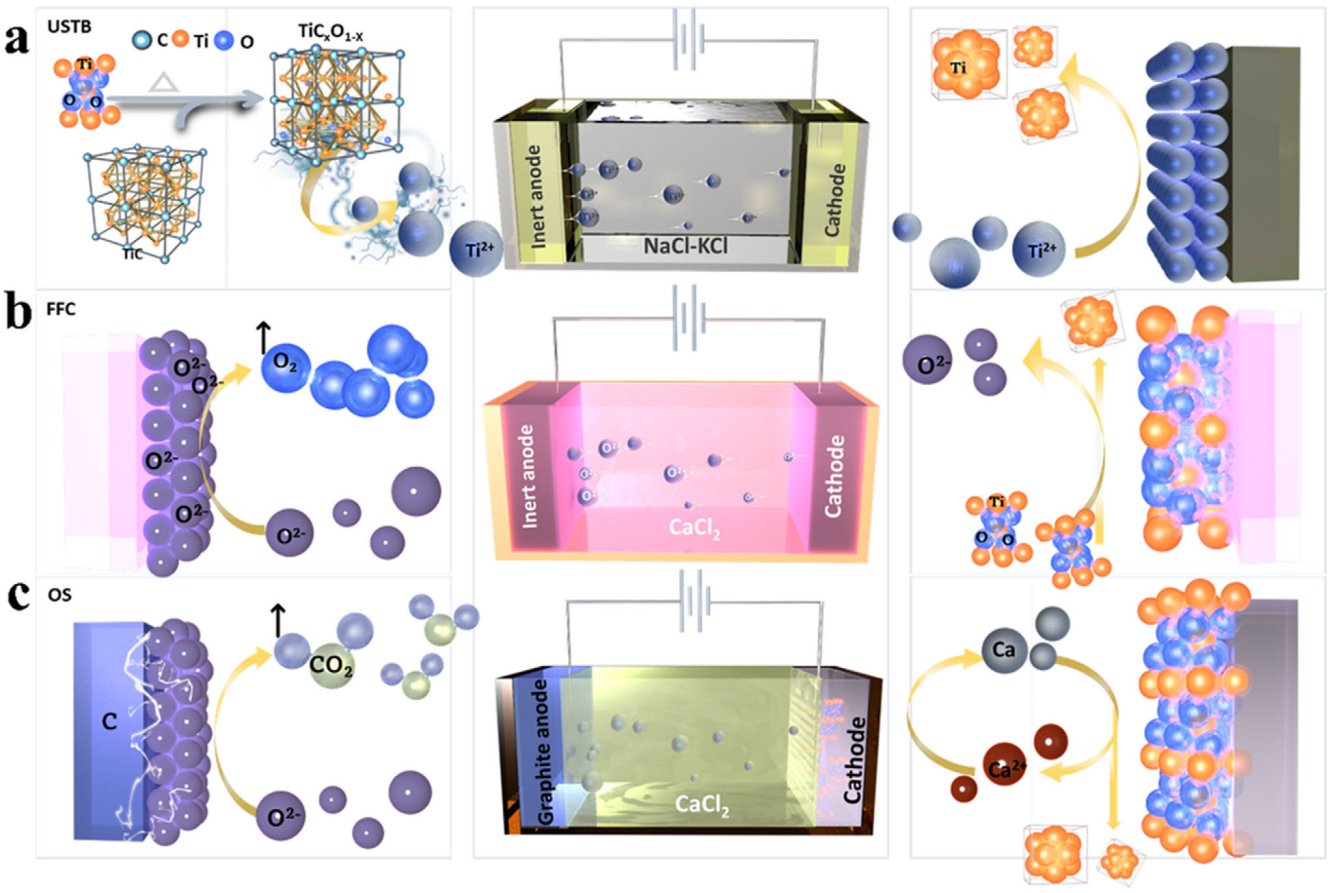
The schematic diagrams illustrate the principles of a) USTB, b) FFC, and c) OS methods, respectively.

Molten salts as the electrolyte exhibits several advantageous features, including high electrochemical stability, excellent thermal stability, high electric conductivity, and low cost.^[^ ^20^ ^]^ Depending on the functional differences of the reaction mechanisms, the high‐temperature molten salt method can be divided into various techniques, including USTB method (**Figure** **3**a),^[^ ^49^ ^]^ the FFC method (Figure 3b),^[^ ^50^ ^]^ OS method (Figure 3c),^[^ ^51^ ^]^ and others. The OS method is similar to the FFC method but relies more on thermal reduction rather than electrolytic reduction, so it is not introduced in this review. The USTB method utilizes metal oxides, such as TiO_2_, mixed with carbon powder or the corresponding metal carbide (TiC) in controlled proportions, followed by high‐temperature vacuum sintering to synthesize a conductive TiC_x_O_y_‐based soluble anode. Under the influence of an electric field, the anode undergoes oxidation, releasing metal ions into the electrolyte, which are subsequently reduced and deposited at the cathode.^[^ ^49^ ^]^ However, this method involves complex raw material preparation, high energy consumption, and significant constraints on in‐situ resource utilization. Therefore, the subsequent discussion primarily focuses on the FFC method.

In 2000, a research team from the University of Cambridge proposed an electrochemical method, known as the FFC process, which enables the direct reduction of solid TiO_2_ in a molten calcium chloride electrolyte.^[^ ^52^ ^]^ During this process, oxygen from TiO_2_ is progressively ionized and migrates into the electrolyte before being discharged at the anode, while pure titanium or its alloy forms at the cathode.^[^ ^53^ ^]^ Currently, two primary mechanisms have been proposed to explain the electrochemical deoxygenation of TiO_2_ in molten CaCl_2_. The first mechanism suggests that calcium is initially deposited at the cathode, where it reacts with oxygen from TiO_2_ to form CaO, which subsequently dissolves in the molten CaCl_2_. The second explanation posits that the cathodic (negative) potential required for oxygen ionization is lower than that for calcium deposition, enabling the direct electrochemical reduction of titanium oxides to metallic titanium without involving a chemical reaction with calcium.^[^ ^54^, ^55^ ^]^ These two deoxygenation concepts can be summarized as follows:

When at higher cathodic potentials, calcium ions will deposit and participate in the reduction reaction:

(1) $$Ca^{2+}+2e^{-}↔\mathrm{Ca}$$

(2) $$\mathrm{Me}O_{x}+\mathrm{xCa}↔\mathrm{Me}+\mathrm{xCaO}$$

When at lower cathodic potential, only oxygen ionization process occurs^[^ ^50^ ^]^:

(3) $$\mathrm{Me}O_{x}+2xe^{-}↔\mathrm{Me}+xO^{2-}$$

**Figure 4:**
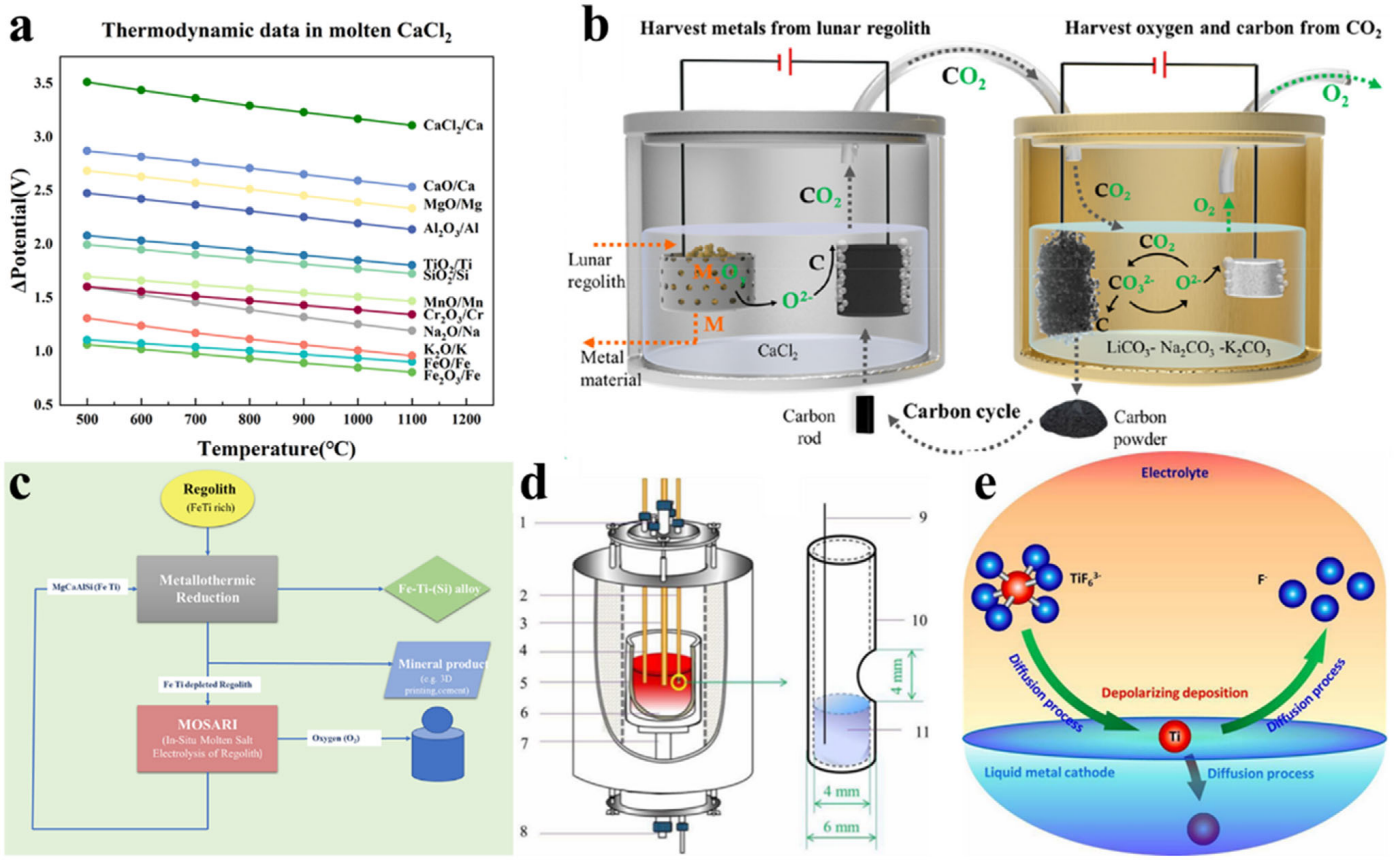
a) Deposition potentials of major oxides in lunar soil in the molten CaCl_2_ system, calculated based on the Gibbs free energy changes derived from HSC Chemistry version 6. b) Schematic diagrams of a molten CaCl_2_ electrolyser and a molten carbonate electrolyser, illustrating the carbon recycling process between the two systems. Reproduced with permission.^[^ ^68^ ^]^ Copyright 2022, American Chemical Society. c) Integrated conceptual design proposed by RWTH Aachen. Reproduced under terms of the CC‐BY license.^[^ ^69^ ^]^ Copyright 2022, The Alexandra Radl et al, published by Springer Nature. d) Structural diagram of the three‐electrode system with a liquid metal cathode. 1. Ar outlet, 2. working electrode, 3. reference electrode, 4. counter electrode, 5. alumina crucible, 6. molten salt, 7. support rod, 8. Ar inlet, 9. molybdenum wire (Φ = 0.4 mm), 10. alumina crucible, 11. liquid metal. Reproduced with permission.^[^ ^74^ ^]^ Copyright 2021, The Electrochemical Society. e) Schematic illustration showing the depolarized deposition process of titanium ions at the liquid metal cathode surface. Reproduced with permission.^[^ ^74^ ^]^ Copyright 2021, The Electrochemical Society.

FFC is characterized by the fact that the reduce reaction takes place at the solid cathode, where the molten salt is not consumed.^[^ ^48^ ^]^ The primary innovation of the FFC is grounded in the thermodynamic principle that the decomposition voltages of various solid oxides at elevated temperatures, specifically between 873 and 1173 K, are lower than those of inorganic molten salts (as illustrated in **Figure** **4**a).^[^ ^50^, ^56^ ^]^ It is important to highlight that ionized oxygen (O^2−^) is transported and remains present in the melt during the process of electro‐deoxidation. Studies have indicated that the discharge of O^2−^ at the graphite anode is thermodynamically more favorable compared to that of Cl^−^. The decomposition of molten chlorides, such as CaCl_2_ or LiCl, during the electrochemical reduction of FFC is influenced by the decomposition voltage of CaO or Li_2_O. It is crucial to consider the varying concentrations (or activities) of O^2−^ and Cl^−^ ions in both the molten CaCl_2_ and at the electrochemical interface. When a suitable cathodic overpotential is applied to the oxide cathodes, solid metals can be produced via the direct electrochemical reduction or electro‐deoxidation of the oxides, as illustrated by reaction (2). This occurs under the condition that both the oxide precursors and their corresponding metals do not significantly dissolve in the molten salts. Simultaneously, the oxygen in the oxides is ionized at the cathode. The ionized oxygen then diffuses from the cathode into the molten salt and is ultimately electro‐oxidized to form oxygen at an inert anode surface, or carbon monoxide (CO) and carbon dioxide (CO_2_) at a carbon anode.

The direct electro‐deoxidation mechanism closely parallels the reduction pathway observed in second‐type electrochemical processes. Previous studies on the electrolysis of solid silica have introduced the initiation–expansion–shrinking–disappearing (IESD) process as a mechanism to describe the reactions occurring at the three‐phase boundary (TPB).^[^ ^57^ ^]^ This boundary involves solid conductive products, solid oxides, and liquid molten salt during the direct electro‐deoxidation of solid oxides.^[^ ^58^, ^59^ ^]^ Given that the metal oxide (MO) is an insulator, a good initial contact between the current collector and the oxide is essential for the onset of oxygen ionization at the cathode. The direct electrochemical reduction of the insulating MO starts at the initial current collector/solid MO/molten CaCl_2_ TPB.^[^ ^60^ ^]^ The reduced metal (M) demonstrates good electrical conductivity and possesses a porous structure due to decreased molar volume. Concurrently, molten CaCl_2_ permeates the pores, leading to the establishment of a new three‐phase boundary (M/MO/CaCl_2_) that extends along the surface and penetrates into the solid metal oxide (MO) until the reduction process is complete. Once the MO is reduced, O^2−^ ions migrate into the surrounding electrolyte, encompassing the reduced, porous metal layer. These ions are subsequently transported to the bulk electrolyte before being discharged at the anode. The advancement of the three‐phase reaction boundary—both along the surface and into the bulk—facilitates the comprehensive electrochemical reduction of the oxides.^[^ ^58^, ^59^, ^61^ ^]^ The kinetics of the electrochemical reduction process occurring at an oxide cathode can be elucidated through the application of a dynamic three‐phase reaction boundary model. This model encompasses the interactions between solid conductive products, solid oxides, and liquid molten salt, thereby providing a comprehensive framework for understanding the underlying mechanisms of the reaction.^[^ ^47^, ^48^, ^49^ ^]^

The electrochemical reduction of solid oxides that feature a single thermodynamically stable metal‐oxygen (M–O) phase or exist in low‐valence states can be effectively achieved through a one‐step electro‐deoxidation process. However, many metal‐oxygen binary systems display multiple thermodynamically stable M–O phases. This indicates that precursor oxides may undergo electro‐reduction either in a stepwise manner (if the M–O binary systems consist of multiple‐phase oxides) or continuously (if the M–O binary systems can form solid solutions). A series of oxygen ionization steps can lead to the formation of partially reduced oxides prior to the complete metallization of the cathode. The occurrence of these partially reduced oxides during the direct electrochemical reduction of the oxide at the cathode has been confirmed in the cases of solid Nb_2_O_5_ and TiO_2_ in molten CaCl_2_.^[^ ^62^, ^63^ ^]^ The porosity of the solid oxide cathode during electrochemical reduction is a critical factor to consider. Increased electrode porosity enables more rapid diffusion of O^2−^ ions and minimizes solution resistance. Furthermore, it is essential to assess the metal‐to‐oxide molar volume ratio to optimize the porosity of the feedstock. When this ratio approaches unity, the resulting metal layer may have insufficient porosity, which can impede the effective diffusion of O^2−^ ions.^[^ ^64^ ^]^ This kinetic barrier then can be overcome or bypassed by utilizing a highly porous precursory pellet.^[^ ^65^ ^]^

The continuous or stepwise electro‐deoxidation process also occurs during the molten salt electrolysis of mixed solid oxides. The inherent variation in decomposition voltages among different oxide components leads to a preference for the initial reduction of the less thermodynamically stable oxides. The metal generated at the outset is dispersed throughout the entire cathode, which not only enhances the overall electrical conductivity but also facilitates the formation of additional electrochemically active sites known as triple phase boundaries (TPB). This indicates that the initially formed metal functions as a depolarizer, thereby facilitating the rapid electrochemical reduction of other oxides.^[^ ^65^ ^]^ A notable example is the titanium‐nickel alloy, where nickel is produced first during the initial stages of electrolysis. The complete formation of the TiNi alloy occurs within 4 h. In comparison, a titanium dioxide pellet of identical size and structure necessitates ≈10 h for complete processing. As a result, the optimal current efficiency surpasses 80%, with energy consumption as low as 6 kWh per kilogram of TiNi produced.^[^ ^66^ ^]^

During electro‐deoxidation, several critical factors—such as electrolysis duration, temperature, current, and other reaction conditions—directly influence the efficiency and extent of reduction. A prolonged reaction time allows for more complete deoxidation, while an elevated temperature accelerates the process. Conversely, a lower average current results in a slower reaction rate. Through systematic evaluation of these parameters, optimal reaction conditions can be established. Notably, the FFC process in molten CaCl_2_ has demonstrated the capability to efficiently reduce all metal oxides present in lunar regolith.^[^ ^67^ ^]^ Building upon this foundation, Shi et al. applied the FFC method to develop a system for reducing simulated lunar soil from the Chang'e 5 mission. In their experimental setup, CaCl_2_ served as the electrolyte, with graphite rods functioning as the anode. At the cathode, lunar regolith underwent electrochemical reduction, yielding the desired metallic products. Simultaneously, the oxygen released at the anode reacted with the graphite to form CO_2_, which was subsequently transferred to a secondary electrolytic cell containing a LiCO_3_‐Na_2_CO_3_‐K_2_CO_3_ electrolyte. This secondary cell facilitated the decomposition of CO_2_ into elemental carbon and O_2_, thereby establishing a closed‐loop process that enables the in‐situ recycling of graphite for sustainable lunar resource utilization (Figure 4b).^[^ ^68^ ^]^

Expanding on these advancements, Alexandra Radl explored the broader application of the FFC process for simultaneous oxygen and metal production from lunar regolith. Using chloride or fluoride‐based electrolytes, the process enables the selective deoxidation of metal oxides at the cathode, while oxygen evolution occurs at the anode. Key lunar oxides—including TiO_2_, Fe_2_O_3_, SiO_2_, and Al_2_O_3_—undergo electrochemical reduction, yielding corresponding metallic or alloyed products (Figure 4c). However, the study also identified a potential challenge: the presence of Fe^2+^ in lunar regolith may negatively impact oxygen evolution efficiency, a phenomenon later corroborated in UTE electrolysis studies. These findings underscore the importance of optimizing key parameters—such as electrode materials and electrolyte composition—to enhance oxygen recovery rates and improve the purity of metal products.^[^ ^69^ ^]^

Meurisse et al. further investigated the feasibility of low‐temperature molten salt electrolysis (660–950 °C) for lunar regolith reduction. Using LMS‐1 lunar regolith simulant, their team conducted systematic electrolysis experiments in various molten salt systems (CaCl_2_‐KCl, CaCl_2_‐NaCl, CaCl_2_‐LiCl, and pure LiCl). Among these, the CaCl_2_‐NaCl system at 680 °C exhibited the highest oxygen removal efficiency (40%), the greatest current efficiency (>60%), and minimal side reactions, making it the optimal choice. These findings pave the way for lower‐temperature electrolysis strategies, which could enhance oxygen extraction efficiency and support the development of sustainable in‐situ resource utilization for future lunar bases.^[^ ^67^ ^]^

Finally, to ensure the production of high‐purity oxygen, Schlüter et al. examined gas purification strategies for lunar regolith electrolysis. Their experiments, conducted in CaCl_2_ and NaCl‐KCl molten salts, confirmed that while oxygen evolves at the anode, it may be contaminated with volatile impurities such as Cl_2_ and F_2_. To address this challenge, the study proposed a two‐stage purification process: 1) hot gas adsorption to remove chlorine and fluorine contaminants, followed by 2) cryogenic distillation to further separate oxygen from residual impurities. This strategy significantly improves oxygen purity, making it suitable for applications in propulsion systems and life support technologies, thereby advancing the feasibility of self‐sustaining oxygen production for lunar bases.^[^ ^70^ ^]^

The FFC process, as we have always known, uses a solid cathode in the electrolysis process with the metal ions diffusing to the cathodic surface for electrochemical reduction. The products can be collected at the cathode, and the cathode can be reused after cleaning. Although the solid‐state cathode is straightforward to operate, for metals with high reactivity or solubility, it will result in the re‐dissolution of obtained metal on the solid‐state cathode into the electrolyte. Additionally, FFC processes typically use a solid oxide mixture as cathodes, which exhibits low conductivity and a prolonged electrolysis time requirement, consequently reducing current efficiency.^[^ ^71^, ^72^ ^]^ Furthermore, the reduction of electronegative elements is more challenging when using a solid‐state cathode,^[^ ^73^ ^]^ which results in a more complex electrode reaction step (e.g., the preparation of Ti using the FFC method produces a complex disproportionation of the ions in the lower valence states are not easily reduced^[^ ^65^ ^]^). In electrolysis, the carbon‐based anode is susceptible to reacting with greenhouse gases, thereby failing to meet the criteria for efficient, economical, and environmentally friendly production. To address these issues, the solid cathode can be substituted with a liquid metal as a liquid cathode in the original method.^[^ ^71^ ^]^ For example: Jiao^[^ ^74^ ^]^ and others put forth the concept of utilizing NaCl‐KCl‐KF molten salt electrolysis with a liquid cathode comprising Sn and Pb for the one‐step electrolytic generation of Ti, which addresses the drawback of the intricate process involved in molten salt electrolysis for the refinement of titanium (Figure 4d). This approach eliminates the emission of CO_2_ and substantiates the benefit of employing liquid electrodes, while also offering enhanced prospects for the utilization of space mineral resources in smelting (Figure 4e).

### 2.2 Ultra‐High Temperature Molten Salt Electrochemistry

The FFC method, extensively investigated for its application in producing metals, presents several notable advantages. These advantages stem from the broad electrochemical window afforded by high‐temperature molten salts. Additionally, the elevated operational temperature not only enhances the efficiency of the separation process but also facilitates a seamless transition to subsequent stages of metallurgical operations.^[^ ^19^, ^71^, ^75^, ^76^, ^77^ ^]^ Titanium, an indispensable material for aerospace structural applications, poses significant challenges in its extraction processes. Conventional methodologies employing aqueous solutions or molten salts at temperatures below 1000 °C predominantly yield powdered or dendritic solid products. These morphologies introduce substantial complexities in downstream processing and applications, ultimately reducing overall efficiency.^[^ ^19^, ^78^ ^]^

**Figure 5:**
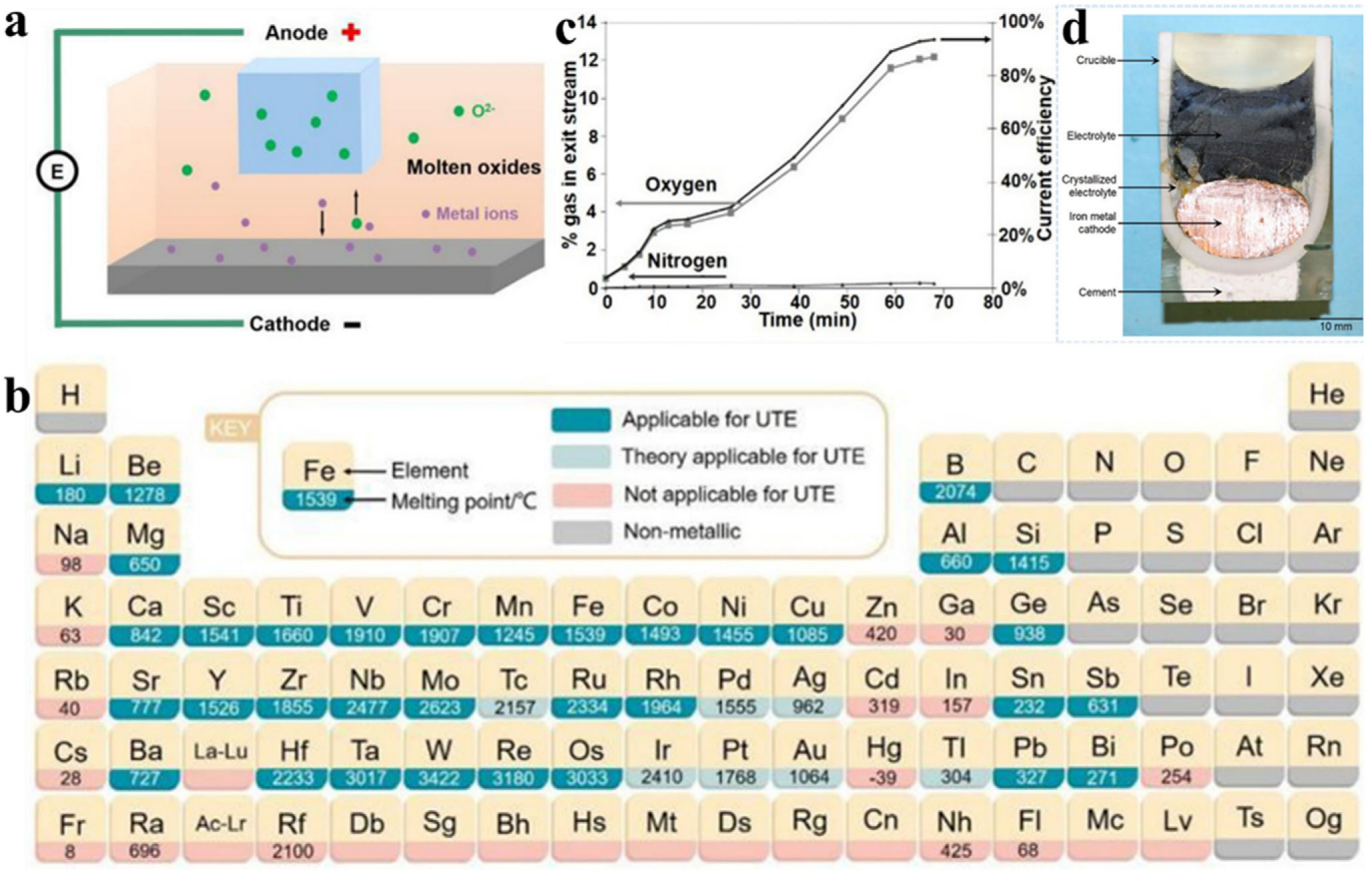
a) Schematic diagram of ultra‐high temperature electrochemistry (UTE). Reproduced with permission.^[^ ^19^ ^]^ Copyright 2022, Wiley‐VCH GmbH. b) Periodic table of elements for UTE. Reproduced with permission.^[^ ^19^ ^]^ Copyright 2022, Wiley‐VCH GmbH. c) Electrochemical cell after the experiment with Fe‐based electrolyte. Reproduced with permission.^[^ ^93^ ^]^ Copyright 2021, The Electrochemical Society. d) Oxygen production from simulated lunar soil via UTE. Oxygen was collected using an anode containment tube; the system operated at 0.2 A with an Ir wire anode, Mo rod cathode, and Pt wire reference electrode. Reproduced with permission.^[^ ^89^ ^]^ Copyright 2021, The Electrochemical Society.

In 1995, Sadoway proposed a transformative approach to the direct electrolysis of metal oxides using chlorides or fluorides as molten salt electrolytes. This innovation obviates the necessity of carbon anodes and simplifies the process by minimizing the number of operational steps. The methodology operates at ultra‐high temperatures (UTE), reaching up to 1550 °C, thereby enhancing process efficiency.^[^ ^79^ ^]^ The UTE concept is predicated on the ability of a molten oxide mixture to dissolve substantial quantities of target oxide feedstocks, operating at temperatures surpassing the melting point of the resultant metal.^[^ ^80^ ^]^ The process predominantly utilizes liquid metal cathodes, such as tin or lithium, which impart a depolarizing effect.^[^ ^74^ ^]^ This effect mitigates the decomposition voltage and substantially enhances the selectivity of the electrochemical reduction process.^[^ ^81^, ^82^ ^]^ Direct electrolysis of molten oxides at elevated temperatures, employing liquid oxides or sulfides as electrolytes, culminates in the production of the target metals.^[^ ^77^, ^83^ ^]^ The comprehensive procedure and applicable elemental range of UTE electrolysis are illustrated in **Figure** **5**a,b.^[^ ^84^ ^]^ Total deposition reaction equation for UTE:

(4) $$\mathrm{Cathodic}\;\mathrm{reaction}:\;\;\;M^{2x+}+2x\;e^{-}=M$$

(5) $$\mathrm{Anodic}\;\mathrm{reaction}:\;\;\;xO^{2-}-2x\;e^{-}=x/2O_{2}$$

(6) $$\mathrm{Aggregate}\;\mathrm{response}:\;\;\;MO_{x}=M+x/2O_{2}$$

Through the UTE method, high‐melting‐point elements such as iron and titanium can be directly extracted from iron ore or titanium slag. This approach mitigates contamination risks inherent in conventional processes that require concentrates or high‐purity metal compounds during the pre‐separation phase. The efficiency and kinetics of electrolysis are contingent upon the temperature and composition of the iron‐containing slags, which vary under different constant current fields.^[^ ^85^ ^]^ Furthermore, UTE enables the production of raw materials amenable to one‐step electrolytic reduction, thereby circumventing multivalent disproportionation a common limitation in traditional processes. For instance, conventional solid‐cathode molten‐salt electrochemical reduction of titanium frequently results in the co‐formation of divalent and trivalent titanium species. By contrast, UTE achieves superior current efficiency and process simplicity, rendering it a highly effective technology. This advancement holds profound implications for space‐related infrastructure, as it significantly reduces energy consumption, thereby offering notable economic advantages.^[^ ^86^ ^]^ In the context of electrochemical reactions, mixed oxide electrolytes facilitate the generation of oxygen at the anode. The resultant oxygen can be directly utilized as a combustion agent or repurposed for life‐support systems in extraterrestrial environments.

At present, UTE has garnered significant attention in the domains of refining and purification, particularly for processing copper sulfide ores, iron ores, and other related materials (Figure 5c).^[^ ^19^, ^87^, ^88^, ^89^ ^]^ Additionally, UTE has proven effective in treating a broad spectrum of metallurgical slags, including those associated with ironmaking, titanium slag, and other complex compositions. For instance, Zou^[^ ^90^ ^]^ proposed the application of UTE for the synthesis of Ti_5_Si_3_. Similarly, Jiao et al.^[^ ^76^ ^]^ demonstrated the feasibility of extracting iron and titanium alloys from oxide feedstocks using a combination of CaO, Al_2_O_3_, and MgO molten salts. Notably, the potential of UTE has been explored in simulated extraterrestrial environments. Lindstrom and Haskin utilized solar energy in conjunction with lunar soil to facilitate direct heating and electrolysis for oxygen production.^[^ ^91^, ^92^ ^]^ D.R. Sadoway et al. extended this research by performing electrolytic reduction at 1500 °C using lunar regolith simulant (JSC‐1), composed primarily of silicon dioxide, iron oxides, aluminum oxide, titanium dioxide, along with MgO, CaO, and other minor oxides, to achieve the simultaneous extraction of metals and oxygen. The feedstock was introduced into a high‐temperature electrolysis reactor, where it was melted into a liquid oxide mixture. During electrolysis, a platinum‐rhodium (Pt‐Rh) anode facilitated oxygen evolution, while a graphite or Pt‐Rh cathode enabled the selective reduction of metals (Fe, Si, Al, Ti), which were subsequently collected. Oxygen ions (O^2−^) migrated toward the anode and were discharged as molecular oxygen (O_2_), whereas metal cations were reduced at the cathode.^[^ ^84^ ^]^ While this approach accommodates protoliths with diverse elemental compositions, its inherent complexity necessitates further optimization. To refine the process, high‐temperature molten salt electrolysis at 1500 °C can be employed, wherein the material is subjected to an external power supply, facilitating controlled oxygen evolution at the anode and selective metal deposition at the cathode.^[^ ^84^ ^]^

Curreri and colleagues investigated the applicability of UTE electrolysis for lunar soil processing. In their study, the regolith was initially melted to form a molten electrolyte, which was subsequently ionized and decomposed into three distinct products: oxygen, a Fe‐Si‐Al‐Ti alloy, and a magnesium oxide‐rich electrolyte. The alloy was then subjected to an electrolytic refining bath for further separation of silicon and various metallic elements. This method effectively addresses the challenges of electrolyzing complex ores to produce oxygen or alloys.^[^ ^93^ ^]^ Nevertheless, the elevated temperatures required for UTE contribute to reduced anode selectivity and incur substantial material costs.

In 2009, Sadoway et al. demonstrated the feasibility of oxygen production from simulated Martian soil through the electrolysis of a SiO_2_‐B_2_O_3_‐Na_2_O mixture with a composition ratio of 0.72:1:1 at 850 °C. This approach highlights the potential of UTE in extraterrestrial applications. Furthermore, intermittent electrolysis of lunar soil using iridium as an inert anode at a current of 5 A for 8 h (in conjunction with JSC‐1) has validated the feasibility of in‐situ fabrication on celestial bodies, achieving simultaneous oxygen and metal production.^[^ ^10^ ^]^ Their team subsequently investigated the efficiency of oxygen extraction via molten electrolysis of lunar regolith simulants with varying iron compositions at 1600 °C. The results demonstrated that in iron‐free melts (devoid of Fe^3+^nd Fe^2+^), the oxygen evolution current efficiency reached 100% (Figure 5c). However, in melts containing Fe^2+^, the efficiency declined to 30%–60%, primarily due to the competing oxidation of Fe^2+^ to Fe^3+^, which reduced the net oxygen yield. Since lunar regolith predominantly contains Fe^2+^ rather than Fe^3+^, these findings provide a more accurate representation of real lunar conditions and highlight the challenges associated with oxygen extraction in practical ISRU applications. Furthermore, the implementation of an anode collection tube significantly improved oxygen evolution efficiency by mitigating oxygen loss and minimizing unwanted side reactions. This study offers a feasibility validation for ISRU‐based lunar oxygen production and establishes a critical foundation for the development of scalable oxygen extraction and metal refining technologies for future lunar exploration (Figure 5d).^[^ ^93^ ^]^ Expanding on these insights, Burke et al. identified additional challenges that electrochemical metal extraction techniques, including the FFC method and UTE method, face in reduced gravity environments. Their research highlights three major obstacles: bubble retention, electrode surface coverage, and reduced electrolysis efficiency. Under lunar or Martian gravity, weakened buoyancy impedes bubble detachment, leading to increased local ohmic resistance, slower electrochemical reaction rates, and, in extreme cases, electrolysis stagnation. Additionally, electrode surface roughness plays a critical role in bubble behavior; rough surfaces promote lateral bubble spreading along the electrode, exacerbating bubble accumulation and further compromising system efficiency.^[^ ^94^ ^]^ To enhance the feasibility of these techniques for ISRU, future research should focus on developing vibration‐assisted bubble detachment, electrode surface engineering optimization, and cross‐flow‐enhanced bubble removal strategies to improve electrolysis stability and oxygen production efficiency in extraterrestrial environments.

While UTE is a highly promising method for resource extraction and metal refining, its process optimization remains a significant challenge. The extreme reaction conditions and the complexity of the electrolytic cell severely hinder real‐time monitoring and control of the electrodeposition process, making systematic improvements both blind and inefficient. Addressing this fundamental limitation, Jiao et al. have developed a groundbreaking multipurpose operando high‐temperature electrochemical instrument that integrates operando Raman microspectroscopy, optical microscopy imaging, and a tunable magnetic field. This innovative system provides real‐time insights into the dynamic behavior of electrode reactions under extreme conditions, enabling a deeper understanding of UTE processes and facilitating rational process optimization. By offering a critical tool for analyzing and refining high‐temperature electrolysis, this work represents a major advancement in the field and holds significant potential for improving the efficiency and scalability of resource utilization technologies.^[^ ^95^ ^]^

## 3 Water Resource Utilization (Water Electrolysis)

While the extraction of metal resources through molten salt electrolysis provides the foundation for constructing extraterrestrial infrastructure, sustaining long‐term human presence in space also hinges critically on the availability and management of water resources—both for life support and for enabling electrochemical energy systems. Recent breakthroughs in extraterrestrial research have challenged the long‐held belief that the Moon is devoid of water, and the uncertain availability of water on Mars. Geological studies, coupled with advancements in detection technologies, have revealed significant new insights into the presence of water on other planetary bodies. Notably, in 2016, NASA's Mars Reconnaissance Orbiter detected substantial amounts of subterranean ice, suggesting that water is not only present but may be accessible in the form of frozen reserves. In addition, a 2018 study by Italian researchers identified subglacial lakes on Mars, further indicating the potential for liquid water beneath the planet's surface.^[^ ^96^, ^97^, ^98^, ^99^, ^100^, ^101^, ^102^, ^103^, ^104^, ^105^ ^]^ These findings, alongside the analysis of meteorite fragments, suggest that extraterrestrial bodies may contain as much as 10% crystalline water, expanding the potential for water‐based resources in space.^[^ ^106^ ^]^

In light of these discoveries, water is increasingly recognized as a critical resource for future space exploration. As Adam Smith once stated, “There is nothing like water,” and this adage holds significant relevance in space, where water can serve as a raw material for propellants, life support systems, radiation shielding, and building materials on the Moon, Mars, and other celestial bodies. The discovery of water in space offers the possibility of a self‐sustaining exploration infrastructure, reducing dependence on Earth‐based supply chains.

Water electrolysis, the process by which water is decomposed into hydrogen and oxygen using an electric current, has long been recognized as a critical technology for energy production. The process was first explored by Troostwijk and Deiman^[^ ^107^ ^]^ in 1789, and by 1948, the first pressurized electrolyzer was manufactured.^[^ ^108^ ^]^ Electrolysis is composed of two half‐reactions: the anodic oxygen‐extraction reaction (OER) and the cathodic hydrogen‐extraction reaction (HER), as represented by the following equation^[^ ^109^ ^]^:

(7) $$\mathrm{Cathodic}\;\mathrm{reaction}:\;4H^{+}+4e^{-}\to 2H_{2}$$

(8) $$2H_{2}O+4e^{-}\to 2H_{2}+4OH^{-}\left(\mathrm{alkaline}\;\mathrm{electrolyte}\right)$$

(9) $$\mathrm{Anodic}\;\;\mathrm{reaction}:\;2H_{2}O\to O_{2}+4H^{+}+4e^{-}$$

(10) $$4OH^{-}\to O_{2}+2H_{2}O+4e^{-}\left(\mathrm{alkaline}\;\mathrm{electrolyte}\right)$$

(11) $$\mathrm{Aggregate}\;\mathrm{response}:\;H_{2}O+\mathrm{direct}\;\mathrm{current}\to H_{2}+\frac{1}{2}O_{2}$$

Hydrogen, as a high‐energy‐density fuel, is particularly valuable in space. When used in propulsion systems, it can achieve energy efficiencies of up to 90%, making it an ideal fuel for spacecraft and lunar/Martian surface operations.^[^ ^110^, ^111^ ^]^ Beyond its role in propulsion, hydrogen also plays a vital role in space‐based metallurgy, where it can serve as a reducing agent for metal extraction. For instance, ilmenite (FeTiO_3_) can be reduced by hydrogen to produce metallic iron and titanium dioxide, releasing water vapor as a byproduct. This water vapor can be condensed and fed back into the electrolysis system, enabling a closed‐loop hydrogen cycle that significantly enhances resource efficiency^[^ ^86^, ^112^ ^]^

Oxygen, the counterpart to hydrogen in electrolysis, is equally indispensable in space. As a primary oxidizer, it enables high‐thrust propulsion in liquid‐fueled rocket engines. Simultaneously, it provides breathable air for astronauts, drastically reducing the reliance on Earth‐based resupply missions and facilitating the development of long‐term space habitats.^[^ ^69^ ^]^

Beyond its critical role in propulsion and life support, electrolysis technology also enables space‐based industrial applications. One promising example is the use of hydrogen in molten salt electrolysis to exfoliate graphite into high‐quality graphene nanosheets, which can be utilized in energy storage systems such as supercapacitors and batteries, essential for sustaining long‐term space operations.^[^ ^113^ ^]^ Given its versatility and efficiency, electrolysis stands out as a key enabler of self‐sustaining space missions. In practice, electrolyzers are designed as modular systems, configured in series or parallel to meet mission‐specific demands. The resulting hydrogen and oxygen must then be carefully cooled, purified, compressed, and stored, ensuring optimal integration into propulsion, life support, and industrial processes.^[^ ^114^ ^]^

**Figure 6:**
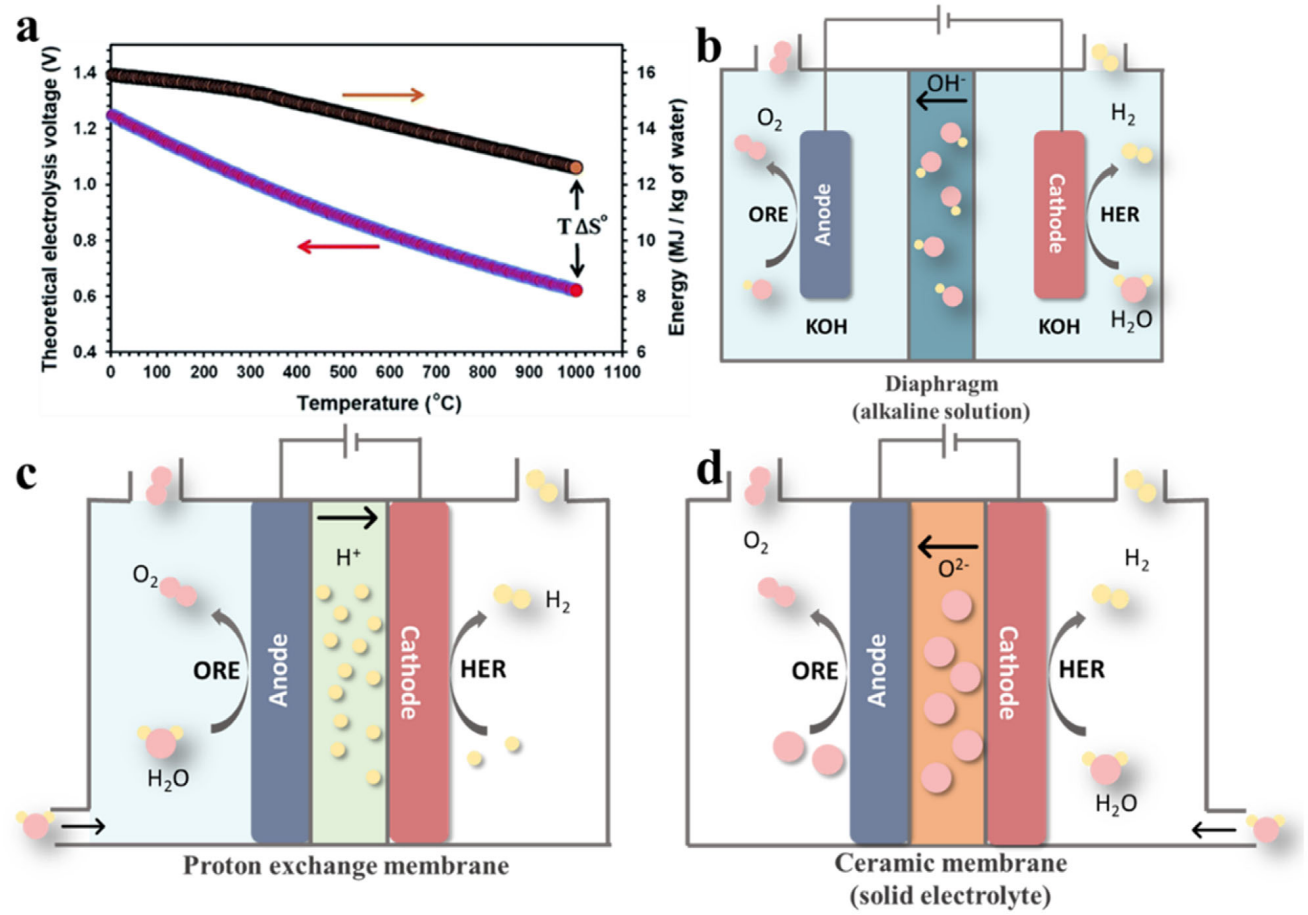
a) Theoretical electrolysis voltage and energy required for the electrolysis of water to produce hydrogen and oxygen. Reproduced under terms of the CC‐BY license.^[^ ^113^ ^]^ Copyright 2020, The Ali Reza Kamali, published by The Royal Society of Chemistry. with permission from the Royal Society of Chemistry. b–d) Schematic diagrams of three types of electrolysers.

Despite the clear potential of electrolysis, practical challenges remain in optimizing the process for space environments. The theoretical minimum voltage required to split water is 1.23 V, but in practice, the threshold voltage is ≈2.0 V due to losses from ohmic resistance, activation overpotentials at the electrode surface, and slow mass transfer in the electrolyte.^[^ ^113^, ^115^ ^]^ To improve efficiency, three primary electrolysis technologies have been developed:, Alkaline Water Electrolysis (AEC) (Figure 6b), Proton Exchange Membrane Electrolysis (PEM) (Figure 6c), and Solid‐State Oxidative Electrolysis Cells (SOEC) (Figure 6d).^[^ ^116^ ^]^ The ongoing development of these technologies aims to reduce the energy consumption of water electrolysis, making it more cost‐effective and suitable for deployment in space missions.

### 3.1 Alkaline Water Electrolysis

**Figure 7:**
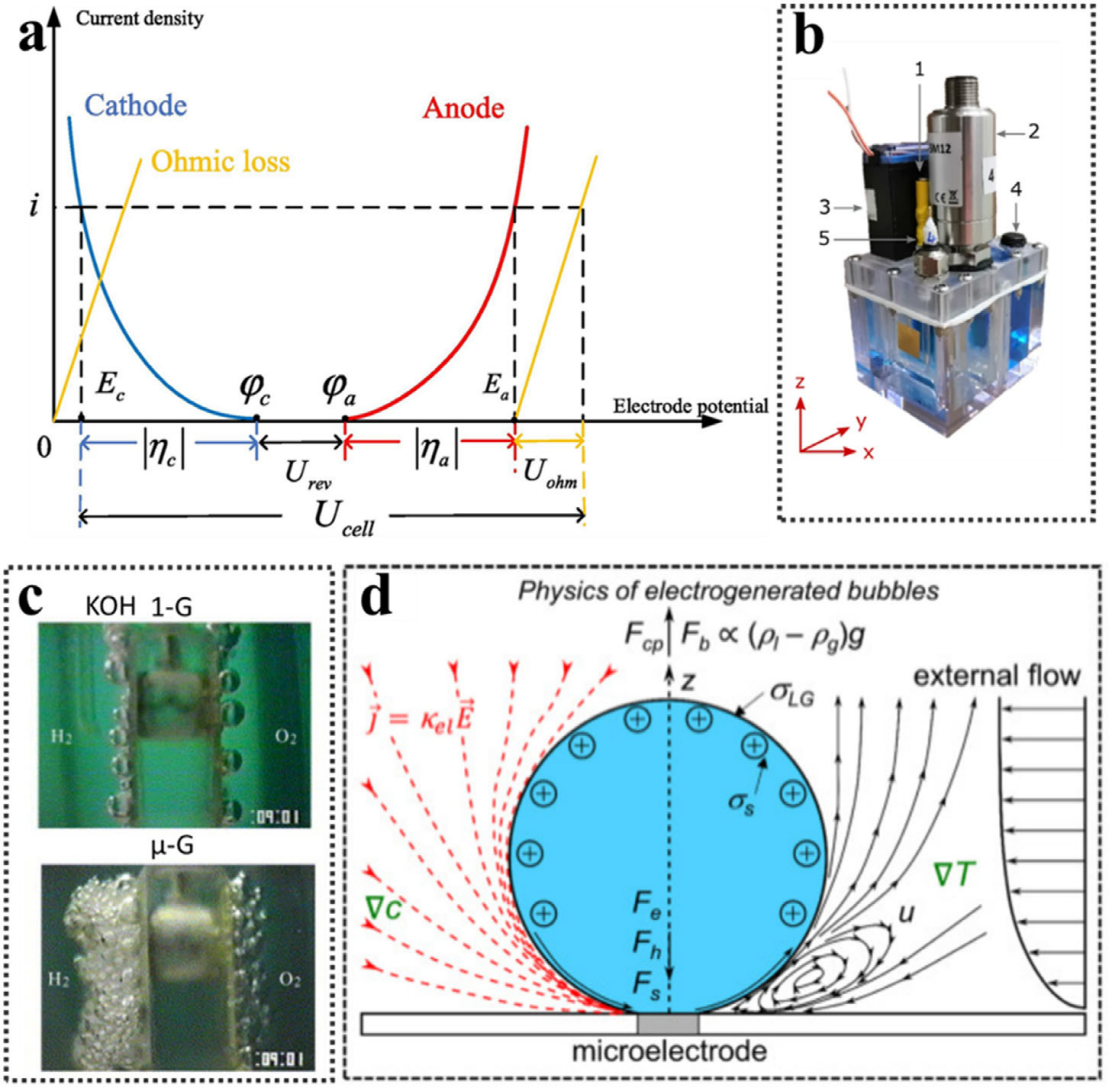
a) Electrode activation characteristics of electrolysis. Reproduced with permission.^[^ ^123^ ^]^ Copyright 2022, Elsevier. b) Polycarbonate electrolysis cell showing: 1) electrode connection, 2) pressure sensor, 3) pressure release valve, 4) epoxy‐filled groove for wire and solder, and (5) polycarbonate electrode holder. Reproduced under terms of the CC‐BY license.^[^ ^127^ ^]^ Copyright 2022, The Behany A. Lomax et al, published by Springer Nature. c) CCD images of gas bubble evolution in 25 wt.% KOH under terrestrial gravity (1‐G) and microgravity (µ‐G), recorded 8 s after the onset of electrolysis (E = −0.8 V versus RHE). Reproduced with permission.^[^ ^129^ ^]^ Copyright 2003, Elsevier. d) Schematic representation of bubble‐associated physical phenomena at microelectrodes. Red lines indicate current pathways from anode to cathode; black lines represent Marangoni convection at the bubble foot. The bubble's surface charge density (σ_s_) is positive below the isoelectric point (IEP), and negative above it. Reproduced under terms of the CC‐BY license.^[^ ^126^ ^]^ Copyright 2022, The Ömer Akay et al, published by Springer Nature.

Although the initial discovery of water electrolysis was made using acidic electrolytes, alkaline water electrolysis (AECs) have since dominated the water electrolysis market due to their relatively lower cost, greater stability, and longer operational lifespan.^[^ ^117^, ^118^ ^]^ An AEC consists primarily of two electrodes, separated by a gas‐tight diaphragm, and is typically operated in a liquid electrolyte, such as a high‐concentration potassium hydroxide (KOH) solution, which ensures high ionic conductivity and enhances system efficiency.^[^ ^119^, ^120^, ^121^ ^]^ The anode is commonly made of nickel or nickel‐plated steel,^[^ ^122^ ^]^ whereas the cathode typically consists of activated steel coated with catalytic materials to enhance reaction efficiency. In a water electrolysis system, electrode activation is crucial, as electrochemical reactions can only occur when sufficient overpotential is supplied to the electrodes. Specifically, the activation overpotential elevates the potential of the anode and reduces the potential of the cathode, thus determining the electrochemical reaction rate and the resultant hydrogen production efficiency. However, increased activation overpotential leads to higher energy losses, thereby negatively influencing the system's overall efficiency (**Figure** **7**a).^[^ ^123^ ^]^

Alkaline electrolyzers generally operate with a cell voltage of ≈2 V and current densities ≈300 mA cm^−2^, achieving a voltage efficiency of ≈70%. The energy consumption of AECs is influenced by the electrode materials and the conditions under which the system operates. Under atmospheric pressure, the energy required for hydrogen production is typically ≈4.1–4.5 kWh per normal cubic meter (Nm^3^) of hydrogen. Under higher pressure, this energy requirement remains similar, though the compression energy for hydrogen is reduced, which is an advantage for space applications, where energy resources are highly constrained.^[^ ^109^, ^118^, ^124^, ^125^ ^]^

A key advantage of AECs is their durability. The high stability of nickel‐based catalysts in alkaline environments ensures that the electrolyzers have a long operational life—typically exceeding 20 years. This longevity, combined with relatively low operational costs, makes AECs an attractive solution for space‐based applications, where maintenance over extended periods is crucial. Recent innovations have explored more advanced configurations, such as a “zero‐gap” setup featuring catalysts and porous transport layers (PTLs), as well as using dual membranes instead of a single diaphragm.^[^ ^126^ ^]^ In this design, the electrolyte flows between the membranes, while gases are separated into distinct chambers, thus preventing the mixing of liquid electrolyte and gas and simplifying the gas collection process.

While these advancements improve the system's efficiency, certain adaptations are required for microgravity environments, such as those encountered in space. In microgravity, the absence of buoyancy alters the behavior of both the electrolyte and the gas bubbles, which in turn impacts the flow dynamics and reaction rates. Lomax et al. demonstrated that under lunar (0.166 g) and Martian (0.376 g) gravity conditions, oxygen evolution efficiency decreases by 6%–11% compared to terrestrial gravity (1 g). This decline is primarily attributed to bubble retention on the electrode surface, which reduces the effective reaction area and impairs overall electrolysis performance (Figure 7b).^[^ ^127^ ^]^ As a result, the design of electrolyzers, including the electrode structure, feed mechanisms, and separator characteristics, needs to be adapted to accommodate the unique conditions of space. Furthermore, PTLs and flow rate systems must be optimized to address the challenges posed by microgravity.

AECs can also operate under high gas pressures, ranging from 6 to 200 bar. High‐pressure operation reduces the energy required for hydrogen compression, which is a significant advantage in space missions, where energy efficiency is critical. However, increasing pressure also reduces the barrier effect of the diaphragm, potentially leading to leakage or loss of efficiency. The use of asbestos mesh for the diaphragm in conventional AECs limits the maximum operating temperature to ≈80 °C, which poses a challenge in the extreme thermal environments of space. The reduced current density, resulting from the less compact design of the electrolyzer, and the decreased effective surface area of the electrodes due to gas bubble formation on their surfaces, also negatively affect the reaction rate. These factors further limit the overall efficiency of the system.^[^ ^109^, ^128^ ^]^ These limitations are particularly pronounced in space, where thermal regulation and energy efficiency are of paramount importance.

The inherent limitations of AECs, such as lower current density and reduced efficiency resulting from gas bubble formation, pose significant challenges for space‐based applications. Under microgravity conditions, these limitations are exacerbated due to enhanced gas bubble retention, coalescence, and impeded detachment. As illustrated in Figure 7c, in terrestrial gravity conditions (upper image), bubbles easily detach and rise from the electrode surface in a KOH electrolyte. In contrast, under microgravity conditions (lower image), the significantly weakened buoyancy causes bubbles to accumulate densely, forming a persistent froth layer on the electrode surface. This bubble accumulation hinders ion transport, increases local overpotential, and consequently reduces the evolution rates of both oxygen and hydrogen, thereby substantially impairing overall electrolysis efficiency.^[^ ^129^ ^]^ Additionally, in microgravity conditions, bubble detachment primarily relies on diffusion and shear flow rather than gravity‐driven buoyancy, leading to a reduced bubble removal rate.

To further understand the physicochemical mechanisms governing bubble behavior in AECs, Yang et al. illustrate the dynamics of electrogenerated bubbles on microelectrodes. The red lines depict the electric current pathways directed from the anode to the cathode, while the black lines schematically indicate the Marangoni convection occurring at the bubble foot. In an electrolyte with a pH value lower than the isoelectric point, the surface charge density of the bubble (σ_S_) is positive; conversely, when the pH exceeds the isoelectric point, the surface charge density becomes negative (Figure 7d).^[^ ^126^, ^129^, ^130^ ^]^ These charge variations influence bubble interactions with the electrode surface and surrounding electrolyte, further affecting bubble detachment and gas evolution efficiency.

To address these challenges and enhance the performance of AECs in microgravity, several optimization strategies can be employed, including the use of nanostructured electrodes or superhydrophobic coatings to reduce bubble adhesion, the introduction of external fluid shear forces or electromagnetic fields to promote bubble detachment, and the application of pulsed current electrolysis to mitigate bubble accumulation on electrode surfaces. These strategies collectively improve the adaptability of AECs for space applications, providing essential technological advancements for oxygen and fuel production in future deep‐space missions.^[^ ^129^, ^131^ ^]^

### 3.2 Proton Exchange Membrane Method

The proton exchange membrane (PEM) system is based on the concept of solid‐state polymer electrolysis, which was first introduced by General Electric in 1960.^[^ ^118^, ^132^ ^]^ Unlike AEC technology, the PEM electrolyzer primarily utilizes a Nafion membrane (a polymer) as the solid electrolyte, with a catalyst coated on the membrane acting as the membrane electrode.^[^ ^133^, ^134^ ^]^ During electrolysis, water is oxidized at the anode to produce oxygen, electrons, and protons. The protons traverse the membrane to the cathode, where they are reduced to form hydrogen, thus completing the electrolysis cycle. Since PEM electrolysis does not rely on a liquid electrolyte, it can achieve a current density of up to 1000 mA cm^−2^. The reduced gas permeability of the polymer electrolyte membrane enhances the purity of hydrogen produced (often exceeding 99.99%, and in some studies reaching 99.999%) and reduces the risk of forming a flammable gas mixture.^[^ ^117^ ^]^

PEM electrolysis typically operates at temperatures ≈80 °C, with pressures reaching up to 15 bar. Standard PEM electrolyzers have capacities ranging from 0.06 to 30 Nm^3^ H_2_/hr, with specific energy consumption in the range of 6–8 kWh/Nm^3^ H_2_. However, at large‐scale production, the specific energy consumption can drop below 6 kWh/Nm^3^ H_2_, yielding efficiencies between 65% and 82%.^[^ ^135^, ^136^ ^]^ Notably, PEM electrolysis cells can operate with variable power supplies, owing to the relatively high inertia of ions within the polymer membrane. This contrasts with the more sluggish ion transport in alkaline electrolysis systems, making PEM technology more responsive to fluctuations in current.^[^ ^116^ ^]^

**Figure 8:**
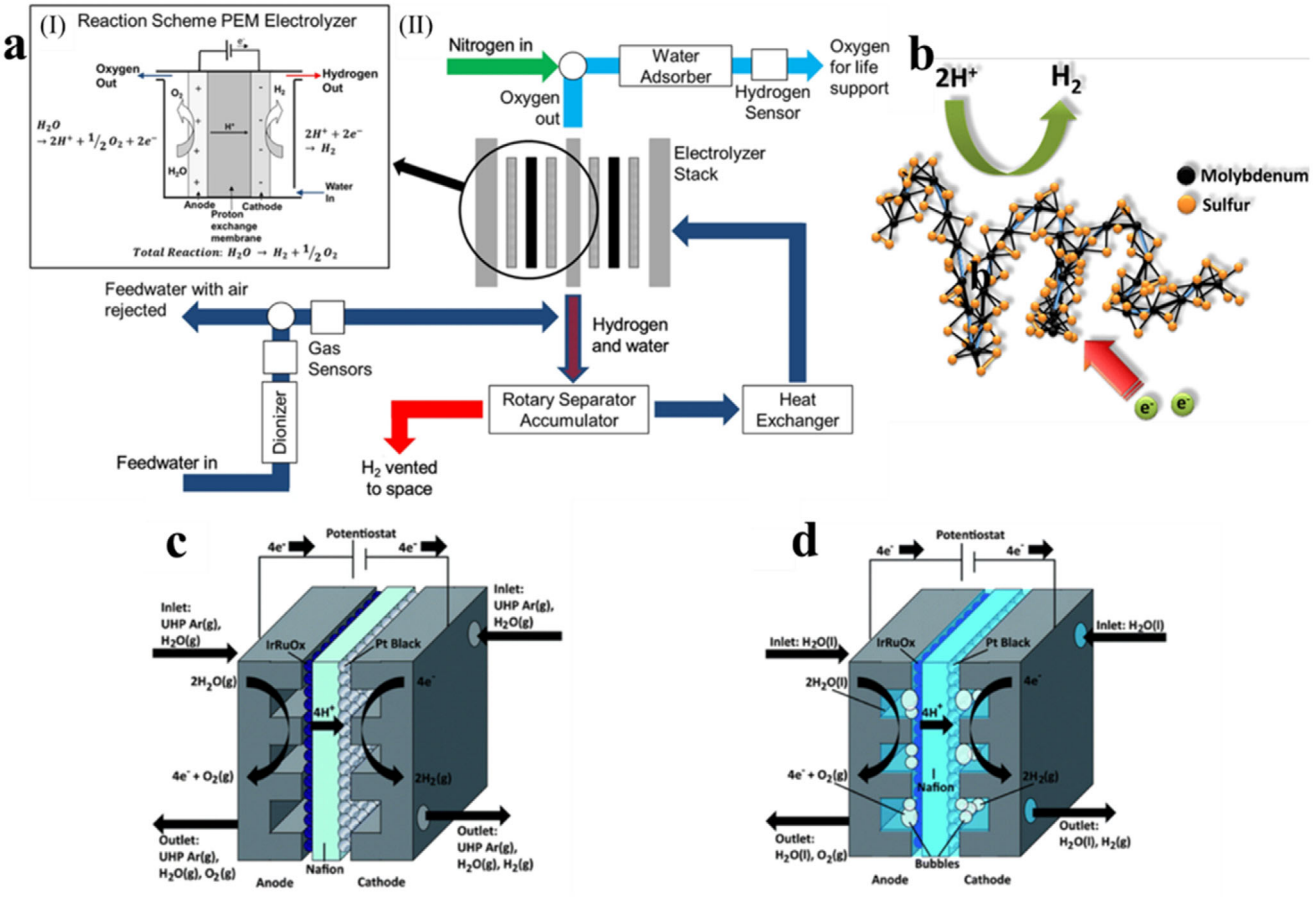
a) Design of a PEM electrolyzer cell (cathode‐feed, inset) and (II) the integration into the Oxygen Generator Assembly (OGA) on the ISS. Reproduced under terms of the CC‐BY license.^[^ ^126^ ^]^ Copyright 2022, The Ömer Akay et al, published by Springer Nature. b) Schematic diagram illustrating the catalytic principle of amorphous molybdenum sulfide for hydrogen evolution. Reproduced with permission.^[^ ^139^ ^]^ Copyright 2014, American Chemical Society. c) Cross‐sectional schematic of the electrolyzer operating with water vapor in ultra‐high purity (UHP) Ar carrier gas as the feedstock. Reproduced with permission.^[^ ^140^ ^]^ Copyright 2011, Royal Society of Chemistry. d) Cross‐sectional schematic of the electrolyzer operating with liquid water as the feedstock. Reproduced with permission.^[^ ^140^ ^]^ Copyright 2011, Royal Society of Chemistry.

Davenport has highlighted that static‐feed PEM electrolyzers are capable of meeting the oxygen and hydrogen demands of space missions, such as the International Space Station (ISS) and future advanced space missions.^[^ ^137^ ^]^ The design and operational parameters of PEM electrolysis systems on the ISS, although not extensively published, have been summarized by Akay et al. based on preliminary ground‐based experiments. These systems include 13 cell stacks, each with an effective area of 214 cm^2^. The cathode inlet water flow rate is 8.07 kg hr^−1^, with an inlet temperature of 339 K, resulting in an average stack temperature of 335 K. The average system pressure is 2860 kN m^2^, with a slight overpressure on the anode side (**Figure** **8**a).^[^ ^126^ ^]^ Platinum‐based catalysts (Type E‐50) are used on the anode, while black platinum is employed on the cathode. These PEM electrolyzers are capable of producing between 2.3 and 9.2 kg of oxygen per day under continuous operation.^[^ ^138^ ^]^

From an energy consumption perspective, improvements to the Oxygen Generation Assembly (OGA) are of particular interest, given its substantial power demand (≈33% of the total energy requirements of the Environmental Control and Life Support System, ECLSS). Furthermore, the acidic operating conditions in PEM electrolyzers necessitate the use of corrosion‐resistant materials, often involving precious metals like platinum for the cathodic catalyst and ruthenium‐iridium‐based oxides for the anodic catalyst.^[^ ^124^, ^139^ ^]^ While new, less expensive catalysts are being developed, such as amorphous molybdenum sulfide catalysts (Figure 8b),^[^ ^139^ ^]^ the challenge of maintaining high current densities and performance at elevated operating potentials and temperatures remains significant. Additionally, the absence of a liquid phase in PEM electrolysis reduces gas evolution but can lead to bubble accumulation between the Nafion membrane and the electrode, further complicating the electrolysis process (Figure 8c,d).^[^ ^109^, ^140^ ^]^

### 3.3 Solid State Oxidation Electrolysis Cells (SOEC)

As previously discussed, water can be oxidized and reduced into hydrogen and oxygen using an electric current at room temperature. However, this process requires a high voltage of ≈2.0 V, making it economically impractical for large‐scale applications. To reduce the overpotential and lower costs, two primary approaches are commonly adopted: 1) developing new electrocatalysts with higher efficiency, and 2) increasing the electrolysis temperature, which lowers the applied voltage. The latter approach is based on the premise that thermal energy can partially replace electrical energy in driving the decomposition of water. While developing high‐performance catalysts often involves expensive raw materials and hazardous chemicals, which are not ideal in space environments, raising the temperature to lower the voltage offers a simpler, more efficient solution that can also capitalize on lunar solar energy. Figure 6a illustrates the theoretical values of voltage and energy required for electrolysis from 0 to 1000 °C. At higher temperatures, it is thermodynamically more favorable to increase thermal energy, thereby reducing the molar Gibbs free energy (∆G) of the reaction while maintaining the molar enthalpy (∆H) constant.^[^ ^141^ ^]^

The most prominent method for high‐temperature water electrolysis is Solid Oxide Steam Electrolysis (SOSE), which operates in the range of 700 to 1000 °C. SOSE, also referred to as high‐temperature steam hydrogen production, is a distinctive hydrogen production technology based on Solid Oxide Electrolysis Cells (SOEC). In this process, water is first heated to generate steam, which is then introduced into the electrolytic cell. At the cathode, water vapor is adsorbed and dissociates into hydrogen ions (H^+^) and oxygen ions (O^2−^). The hydrogen ions combine with free electrons in an external circuit to produce hydrogen gas, while the oxygen ions pass through the solid oxide electrolyte and undergo oxidation at the anode, producing oxygen gas. SOEC systems require solid electrolytes, typically composed of ceramic materials that incorporate rare‐earth element oxides such as barium, cerium, yttrium, ytterbium, and lanthanum. An example of such a material is Ba_0.5_Sr_0.5_Co_0.8_Fe_0.2_O_3‐δ_ (BSCF), which is widely used as an anode material in SOECs due to its high catalytic activity. The production of these materials involves high‐temperature ceramic processing techniques, such as casting and screen printing. These processes can induce significant stress in the crystalline structure, leading to macroscopic cracks that may result in material failure. To mitigate this, Wei et al. conducted simulations to identify the key factors affecting the formation of these cracks and concluded that yttrium‐stabilized zirconium oxide (YSZ) exhibits strength and long‐term reliability under operating conditions.^[^ ^142^ ^]^

**Figure 9:**
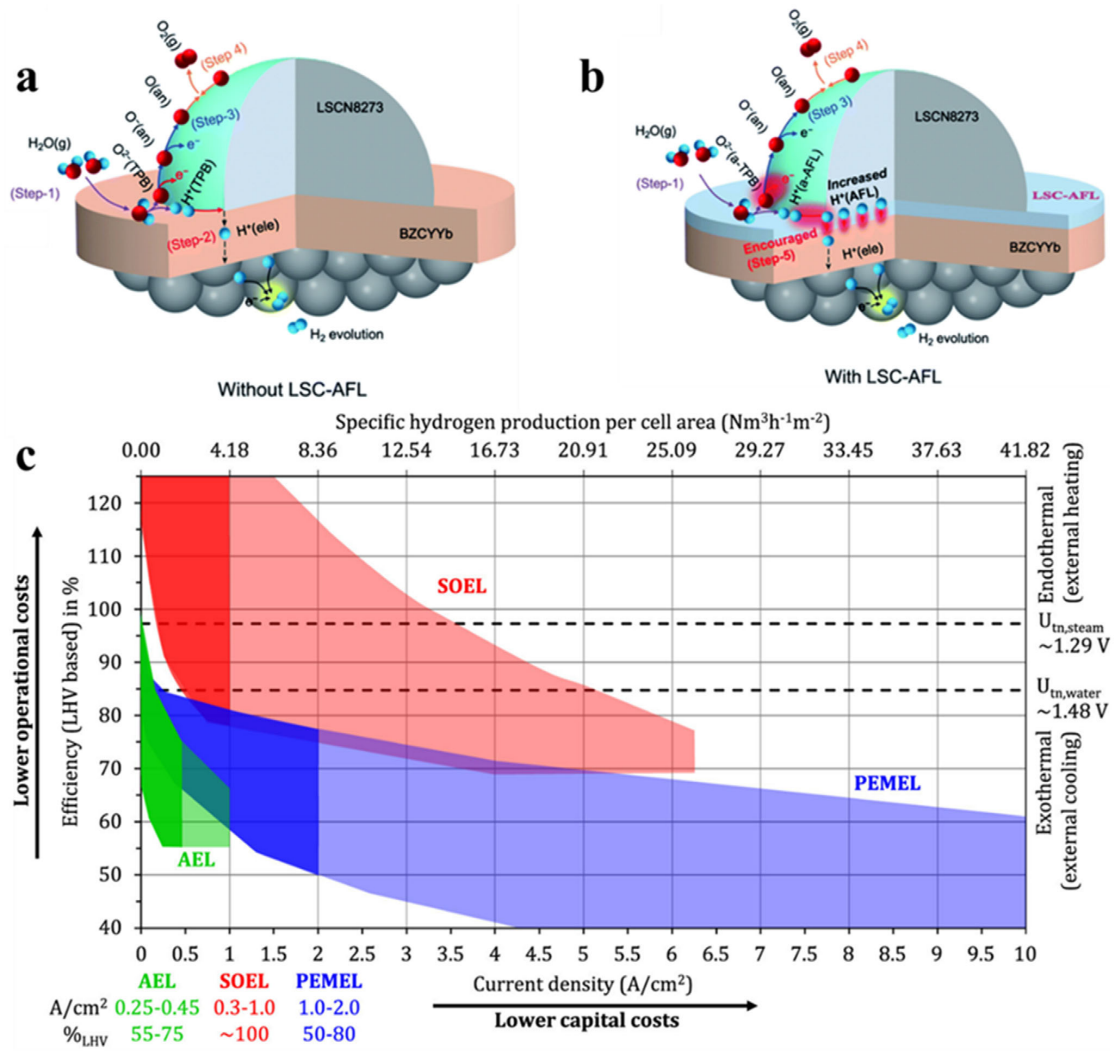
a) Schematic of the anode reaction mechanism in P‐SOECs without LSC‐AFL at the triple‐phase boundary. Reproduced with permission.^[^ ^143^ ^]^ Copyright 2021, Royal Society of Chemistry. b) Schematic of the anode reaction mechanism in P‐SOECs with LSC‐AFL at the triple‐phase boundary. Reproduced with permission.^[^ ^143^ ^]^ Copyright 2021, Royal Society of Chemistry. c) Summary of efficiency and operational ranges of alkaline electrolysis (AEC), proton exchange membrane electrolysis (PEMEL), and solid oxide electrolysis (SOEL) systems. Reproduced with permission.^[^ ^133^ ^]^ Copyright 2018, Elsevier.

Conventional SOEC systems use oxide ionic conductors, such as YSZ, which demonstrate high ionic conductivity at elevated temperatures (700–1000 °C). However, these systems face several challenges, including hot cell degradation, material corrosion, and difficulties related to thermal management. To address these challenges, a new variant known as Proton‐conducting Solid Oxide Electrolytic Cells (P‐SOEC) has been developed. This technology is based on proton‐conducting chalcogenides, such as Ba(Ce,Zr,M)O_3‐δ_ (where M = Y, Yb, etc.). For example, BaCe_0.55_Zr_0.30_Y_0.15_O_3‐δ_ electrolytes have demonstrated peak power densities (PPDs) of 1.3 and 1.1 W/cm^2^, respectively, at 600 °C. Additionally, BaZr_0.4_Ce_0.4_Y_0.1_Yb_0.1_O_3‐δ_ (BZCYYb4411) achieved a peak power density of 1.065 W/cm^2^. To further enhance the performance of P‐SOECs, the incorporation of an anode functional layer (AFL) has been explored. Materials such as La_0.5_Sr_0.5_CoO_3‐δ_ (LSC) have shown significant potential in improving the electrochemical efficiency of these systems by facilitating charge transport and optimizing electrode reactions (**Figure** **9**a,b).^[^ ^143^ ^]^ P‐SOEC systems, with their relatively low conduction activation energy (≈0.5 eV), operate efficiently in the intermediate temperature range (400–600 °C), thereby avoiding the high‐temperature limitations of O‐SOEC and presenting a promising alternative.^[^ ^144^, ^145^, ^146^, ^147^ ^]^

Despite these advancements, the cost of electrode materials remains high. To reduce costs, a novel approach based on high‐temperature molten salt electrolytes for hydrogen production has recently been proposed. This method leverages molten salt systems, such as LiCl, which absorb water in high‐temperature environments through a hydrolysis mechanism, producing hydrogen ions and oxygen ions.

In short, alkaline water electrolysis using aqueous potassium hydroxide is the most commercially mature and cost‐effective among the various methods of water electrolysis, but it suffers from low current density (0.2–0.5 A cm^2^) and high energy consumption. In contrast, SOEC is the most efficient electrolysis technology, though it remains in the research and development phase. SOEC faces challenges such as material degradation, corrosion resistance, sealing, thermal cycling, and chromium migration. However, it offers significant advantages over low‐temperature systems. Thermodynamically, water vapor electrolysis has several advantages over liquid water electrolysis: the enthalpy (∆rH) for water vapor electrolysis is lower due to the heat of vaporization, and the Gibbs free energy (∆rG) decreases with increasing temperature, which leads to a lower standard voltage (E_0_) because of the change in reaction entropy (∆rS) and the temperature (T). In summary, SOEC are highly efficient (Figure 9c),^[^ ^133^ ^]^ and the U.S. Department of Energy (DOE) has initiated several hydrogen production projects utilizing SOEC technology.^[^ ^114^, ^143^, ^148^ ^]^ However, in microgravity environments, the shielding of electrodes by gas bubbles during electrolysis remains a significant technical challenge. This phenomenon can reduce the active surface area of the electrodes, increase ohmic resistance between them, and induce microscopic convection as a result of bubble motion and coalescence.^[^ ^131^, ^138^, ^149^ ^]^ These issues pose substantial obstacles to the successful application of electrochemical technologies for space exploration.

## 4 Carbon Cycle

During space exploration, crews generate waste materials, including food scraps and metabolites with high organic and salt content, as well as substances that are challenging to dispose of, such as urea. Adults excrete urea at a rate of ≈11.8–23.8 g day^−1^.^[^ ^150^ ^]^ Additionally, carbon dioxide exhaust is expected to be produced in significant quantities in future space‐based manufacturing operations. Considering the high costs associated with space transportation—estimated at ≈200 tonnes of life‐support supplies for a six‐person mission to Mars—recycling waste materials presents a more cost‐effective alternative to direct disposal.^[^ ^151^, ^152^ ^]^ Electrochemical methods for in‐situ waste treatment have emerged as promising approaches to address these challenges, enabling the direct or indirect electrolysis of waste materials via electric currents applied through electrodes. These methods not only mitigate waste accumulation but also convert it into valuable resources. Among the commonly explored techniques are microbial fuel cells (MFCs) for organic matter degradation, SOECs for carbon dioxide capture and conversion, and the urea oxidation reaction (UOR).^[^ ^153^ ^]^ While UOR represents a targeted solution for urea‐rich waste streams, its current application is limited by challenges such as catalyst efficiency and durability. Therefore, the focus of this discussion will center on MFCs and SOECs, which exhibit broader applicability in addressing waste management and energy recovery in space missions.

### 4.1 Organic Matter Conversion

MFCs are a notable category of bioelectrochemical systems (BESs) that directly convert organic waste into electricity through microbially catalyzed anodic reactions and microbial, enzymatic, or abiotic cathodic reactions.^[^ ^154^ ^]^ The concept of bacteria generating electricity was first proposed by Potter in 1911 and later validated by Cohen in 1931, who reported a voltage of 35 V at a current of 0.2 mA using a stacked bacterial fuel cell system.^[^ ^155^ ^]^ Although these publications marked the origins of electromicrobiology, it was not until 1963 that the NASA explored the possibility of recycling human waste into electricity during space flights. In 1990, Habberman and Pommer further advanced the field by reporting the first long‐term operational MFC.^[^ ^156^, ^157^ ^]^ MFCs can process a wide range of microbially degradable organic substrates, including simple molecules like carbohydrates and proteins, or complex mixtures derived from human or animal activities, such as food and agricultural waste. For example, MFCs have been successfully integrated into wastewater treatment systems to remove organic pollutants while simultaneously generating electricity. Similarly, in agricultural waste management, manure has been converted into bioenergy. This flexibility makes MFCs uniquely capable of harnessing electrical energy from soluble organic waste streams while regenerating biomas7.

MFCs comprise several key components: an anode, cathode, electrolyte, separator (or proton exchange membrane, PEM), and an external circuit. At the anode, microorganisms oxidize organic substrates, releasing electrons and protons.^[^ ^158^, ^159^ ^]^ The protons diffuse through the PEM into the cathodic compartment, where they combine with oxygen to generate water via the oxygen reduction reaction (ORR):^[^ ^160^, ^161^, ^162^, ^163^ ^]^

(12) $$O_{2}+4H^{+}+4e^{-}\to 2H_{2}O$$

Oxygen is the most commonly used oxidant due to its abundance and high reduction potential. Most MFC configurations operate under anaerobic conditions, which are essential for bacterial species like Geobacter sulfurreducens, renowned for their efficient electron transfer properties.^[^ ^164^ ^]^ Within the anode chamber, microorganisms decompose complex organic compounds into CO₂ and H₂O, releasing electrons and protons.^[^ ^165^ ^]^ These electrons flow through an external circuit from the anode to the cathode, generating electricity due to the potential difference.^[^ ^166^, ^167^ ^]^

**Figure 10:**
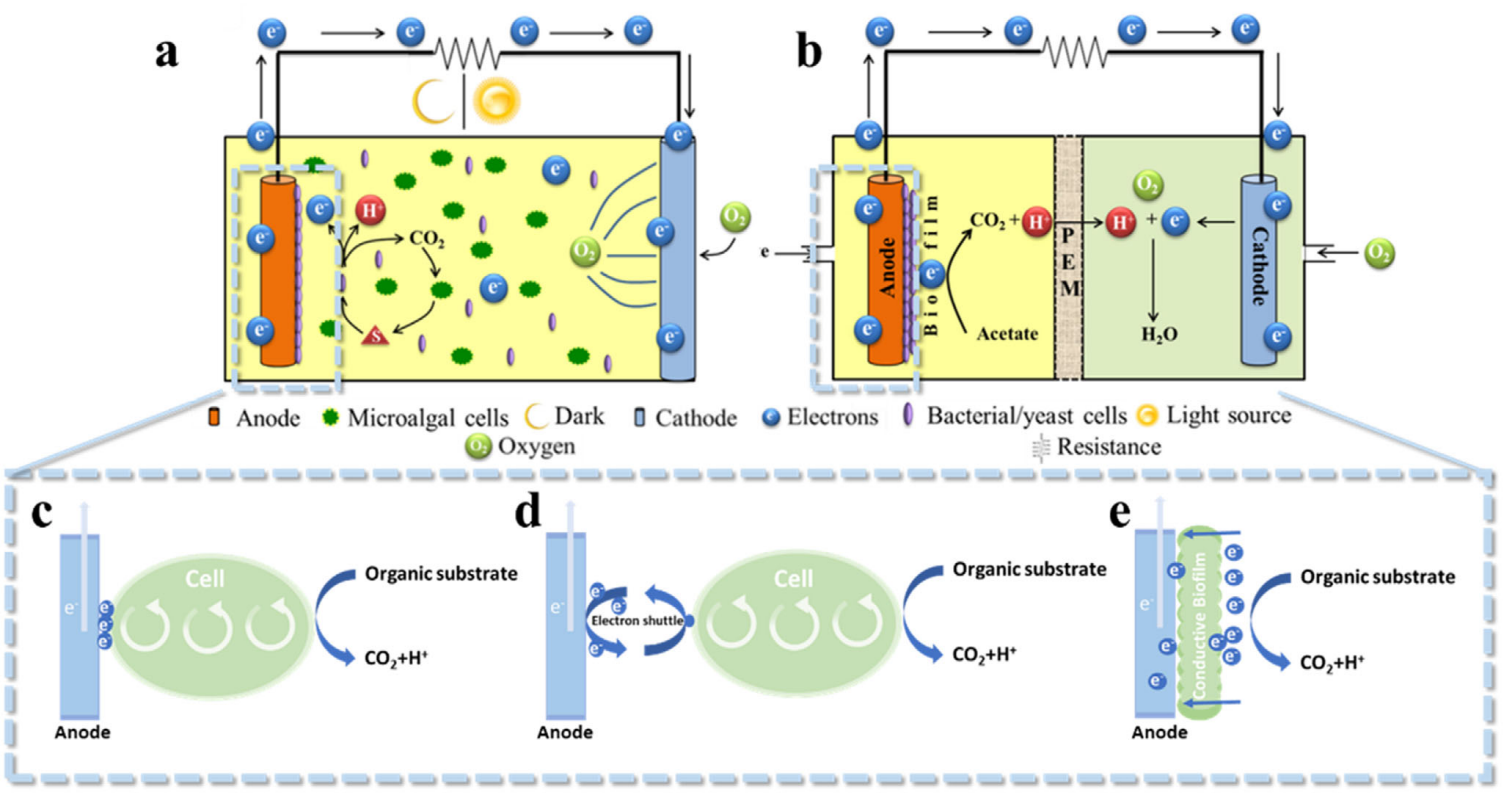
a) Schematic diagram of a single‐compartment photosynthetic microbial fuel cell (PMFC) structure. Reproduced with permission.^[^ ^205^ ^]^ Copyright 2014, Elsevier. b) Schematic structure of a two‐chamber PMFC. Reproduced with permission.^[^ ^205^ ^]^ Copyright 2014, Elsevier. c) Direct electron transfer (DET) model showing microorganisms directly transferring electrons to the anode through conductive nanowires or pili. d) Indirect electron transfer (IET) model showing microorganisms releasing electron shuttles (e.g., phenazines, flavins, or quinones) that transfer electrons to the anode. e) Biofilm‐mediated electron transfer (BMET) model illustrating the formation of conductive biofilms on the anode surface, enabling electron transfer via interconnected conductive structures.

Protons generated at the anode diffuse through the PEM into the cathodic compartment.^[^ ^168^ ^]^ At the cathode, electrons combine with protons and oxygen, leading to water formation via the ORR. Despite the abundance and high reduction potential of oxygen, the ORR remains a significant challenge for MFCs due to high overpotentials and slow kinetics.^[^ ^169^, ^170^ ^]^ This can be calculated as the difference between the standard reduction potentials of the specific anodic substrate and the cathodic oxidant.^[^ ^165^, ^166^, ^171^ ^]^ Alternative oxidants, such as metallic species^[^ ^158^, ^159^ ^]^ (e.g., uranium,^[^ ^172^ ^]^ cadmium^[^ ^173^ ^]^ chromium,^[^ ^174^ ^]^ or copper^[^ ^175^ ^]^), have been explored to address these issues, but their toxicity and reduction requirements pose additional hurdles.^[^ ^158^ ^]^ MFC configurations are categorized into single‐chamber (**Figure** **10**a) and dual‐chamber (Figure 10b) designs based on reactor architecture.^[^ ^176^ ^]^ In dual‐chamber systems, the anode compartment is kept anoxic, while in single‐chamber systems, this is not necessary.^[^ ^170^, ^176^ ^]^ Dual‐chamber systems maintain an anoxic anode compartment to support anaerobic bacteria, whereas single‐chamber systems expose the cathode directly to air, simplifying the design.^[^ ^177^ ^]^ Single‐chamber systems are more financially efficient, as they eliminate the need for a separate cathodic compartment and external electron acceptors. Additionally, passive oxygen transfer to the cathode reduces the energy requirements for air sparging.^[^ ^178^ ^]^ The first reported single chamber MFC was developed in 2003 by Park and Zeikus. Their design used a rubber‐stoppered bottle with a centrally inserted anode and a cathode mounted in a side window. A proton‐permeable porcelain layer was integrated into the cathode, and sewage sludge served as the biocatalyst, achieving a maximum power density of 788 mW m^2^.^[^ ^179^ ^]^ Microorganisms in MFCs utilize three primary electron transfer mechanisms: direct electron transfer (DET) (Figure 10c), indirect electron transfer (IET) (Figure 10d), and biofilm‐mediated electron transfer (BMET) (Figure 10e).^[^ ^180^ ^]^ DET occurs through physical connections between the bacterial cell and the electrode surface, facilitated by nanowires or redox‐active proteins. In contrast, IET relies on electron shuttles produced by bacteria.^[^ ^181^ ^]^ For example, Pseudomonas aeruginosa generates pyocyanin, while Shewanella oneidensis produces quinone mediators, both of which enhance electron transfer efficiency.^[^ ^182^ ^]^ Additionally, BMET involves microorganisms forming conductive biofilms on the electrode surface. These biofilms enable efficient electron transfer through interconnected conductive structures such as pili, nanowires, or extracellular polymeric substances. Prominent examples include Geobacter sulfurreducens, which utilizes conductive nanowires, and Shewanella oneidensis, which relies on c‐type cytochromes within its biofilm matrix.^[^ ^183^ ^]^

The formation of electroactive (EA) biofilms is critical for sustained MFC operation. These biofilms enable efficient electron transfer to the electrode surface. For example, Geobacter sulfurreducens forms biofilms with conductive nanowires,^[^ ^184^ ^]^ while Shewanella oneidensis uses c‐type cytochromes.^[^ ^176^, ^185^ ^]^ EA biofilms form through batch, fed‐batch, or continuous‐flow modes, with fed‐batch being the most common. In this mode, fresh substrates are added or the solution is replenished when electrical output falls below a threshold.^[^ ^186^, ^187^, ^188^ ^]^ The substrates for biofilm formation vary widely, ranging from simple organic molecules like glucose to complex compounds found in agricultural and domestic wastewater.^[^ ^189^, ^190^ ^]^

Beyond MFCs, other bioelectrochemical systems (BESs) such as microbial electrolysis cells (MECs) and microbial desalination cells (MDCs) have been developed.^[^ ^191^, ^192^, ^193^, ^194^, ^195^ ^]^ MECs require an external voltage to produce hydrogen, while MDCs simultaneously treat wastewater, generate electricity, and desalinate water. These systems represent promising advancements but face challenges related to scalability, low reaction kinetics, and cell design.

The theoretical power density of MFCs remains far below its maximum potential. For example, a single Escherichia coli cell, replicating twice per hour with a volume of 0.491 µm^3^, could theoretically produce ≈16 000 kW m^−3^.^[^ ^170^ ^]^ However, actual power densities achieved by current MFC systems are significantly lower due to limitations in microbial activity, electron transfer efficiency, and reactor design.^[^ ^169^, ^171^ ^]^ Addressing these challenges necessitates advancements in electrode materials, optimization of microbial communities, and improved reactor configurations.^[^ ^165^, ^166^, ^171^ ^]^

MFCs face additional challenges related to oxygen reduction at the cathode. The ORR is hindered by high overpotentials and slow reaction kinetics, limiting power output.^[^ ^158^ ^]^ While metallic oxidants such as uranium and copper have been investigated as alternatives, their toxicity and handling complexity reduce their practicality.^[^ ^172^, ^173^, ^174^, ^175^ ^]^ Research into advanced catalysts, such as platinum‐group metals or enzyme‐based systems, offers potential solutions but remains costly.^[^ ^159^ ^]^ MFC applications extend beyond traditional fields such as wastewater treatment and bioenergy production. For example, in space exploration, MFCs could provide a closed‐loop system for converting organic waste, such as urea, into electricity, addressing waste management issues while supplementing energy supplies for spacecraft.^[^ ^156^ ^]^ However, deployment in microgravity environments poses significant challenges, including maintaining stable operation and designing compact systems suitable for space missions.^[^ ^196^ ^]^ Advancements in reactor miniaturization, microbial community optimization, and material development are essential to overcome these obstacles.^[^ ^197^, ^198^, ^199^ ^]^

Despite their potential, MFCs face several limitations, such as low power yields, high operational costs, and scalability challenges.^[^ ^200^, ^201^, ^202^, ^203^ ^]^ Complementary technologies, like anaerobic digestion, provide alternative methods for bioenergy recovery from organic waste. For instance, solid‐state anaerobic digestion enables the treatment of high‐solids‐content food waste on a large scale with minimal energy input, though its energy transfer efficiency remains limited.^[^ ^204^ ^]^ Integrating MFCs with anaerobic digestion or other complementary technologies could improve overall system efficiency and expand their range of applications.

### 4.2 CO_2_ Conversion

Extraterrestrial survival, a cornerstone technology for space exploration, forms the basis for enabling long‐term human activities in space, including Earth and lunar orbital missions, extended Mars missions, prolonged extraterrestrial habitation, and interplanetary migration (e.g., lunar and Martian bases). Achieving these goals requires a sustainable supply of oxygen, fuel, and nutrients over extended periods. Carbon dioxide (CO_2_), a major byproduct of human respiration and some industrial processes, must be efficiently converted into usable resources to establish a closed‐loop carbon cycle. A key challenge in this cycle is capturing CO_2_ from air or exhaust streams, where its partial pressure is often low. The conversion of CO_2_ from human respiration into oxygen within confined spaces can significantly reduce the material resupply needs of manned space stations and deep‐space missions. Additionally, leveraging *in‐situ* resources, such as the abundant CO_2_ and water in extraterrestrial atmospheric environments (e.g., Mars), to produce oxygen and fuel is pivotal for sustaining long‐term human survival on other celestial bodies. This approach also supports deep‐space round‐trip propulsion and transportation, forming the foundation for cost‐effective and sustainable human exploration of deep space. As a result, the in‐situ conversion of H_2_O and CO_2_ is expected to play a crucial role in addressing these challenges.

Electrochemical methods present a promising pathway for such conversions, offering isothermal operation and mitigating thermocatalytic degradation. By precisely controlling reaction processes through potential adjustments, these methods can reduce the energy requirements for electrochemical regeneration of adsorbents.^[^ ^206^ ^]^ Furthermore, the electrochemical utilization of CO_2_, demonstrated in molten carbonate fuel cells, has proven effective for space applications. Two primary electrochemical techniques for carbon cycling are currently utilized in space missions: 1) Direct electrolysis of CO_2_ in solid electrolyte oxygen regeneration systems to produce O_2_ and solid carbon; 2) Reduction of CO_2_ and hydrogen via the Bosch reaction, yielding O_2_, solid carbon, and water, or via the Sabatier reaction, producing methane (CH_4_) and water (H_2_O).^[^ ^207^ ^]^

**Figure 11:**
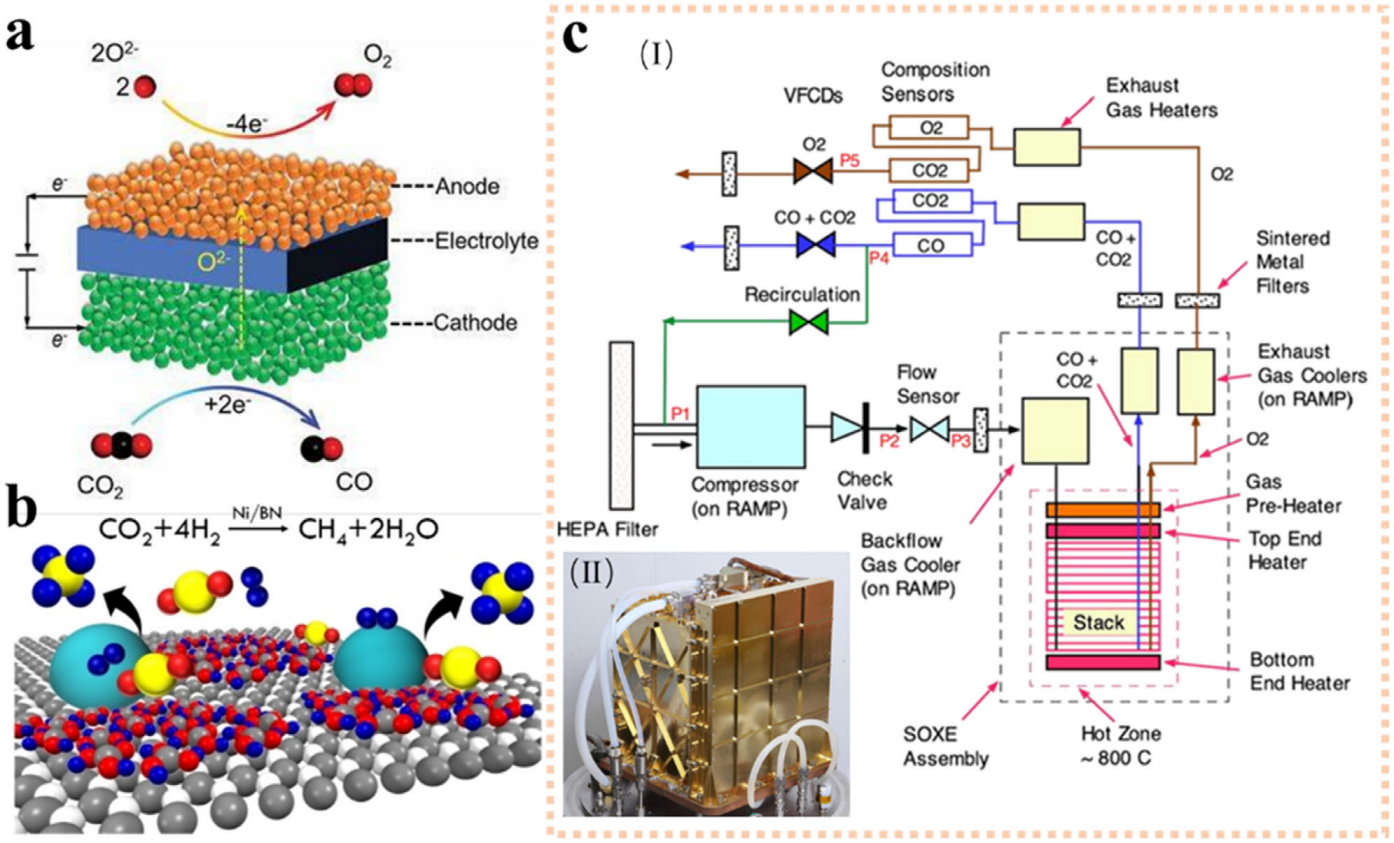
a) Schematic diagram illustrating the CO₂ electrolysis principle. Reproduced with permission.^[^ ^211^ ^]^ Copyright 2019, Wiley‐VCH Verlag GmbH & Co. KGaA, Weinheim. b) Schematic representation of the Sabatier reaction principle. Reproduced with permission.^[^ ^212^ ^]^ Copyright 2023, Wiley‐VCH GmbH. c) I: MOXIE instrument schematic showing compression, electrolysis, and thermal control of the gas streams. The heat exchange unit preheats inlet gas, recovers heat from outlet gas, and blocks reverse flow. A check valve stabilizes pressure, while pressure transducers monitor flow through the orifice. Reproduced under terms of the CC‐BY license.^[^ ^209^ ^]^ Copyright 2021. The Hecht et al., published by Springer Nature. II: The MOXIE instrument onboard NASA's Perseverance rover. Image credit: NASA/JPL‐Caltech/Public domain.

Initially, the Skylab manned spacecraft employed zeolite adsorption beds to recirculate CO_2_. However, the inefficiency of zeolite and its inability to meet the stringent CO_2_ partial pressure requirements in space limited its utility.^[^ ^207^ ^]^ In 1971, Wynveen and Quattrone developed an electrochemical device capable of removing CO_2_ from a vehicle while maintaining cabin air purity during subsequent CO_2_ reduction. By 2003, a fully integrated reactor utilizing both the Sabatier and Bosch reactions was constructed to serve as an oxygen source for space exploration missions.^[^ ^208^ ^]^ In June 2011, the CO_2_ Reduction Assembly (CRA)—the state‐of‐the‐art system for CO_2_ reduction—was deployed on the International Space Station. This system employs the Sabatier reaction to convert CO_2_ into CH_4_ and H_2_O (**Figure** **11**a,b).^[^ ^126^ ^]^

(13) $${\mathrm{CO}}_{2}\;\mathrm{Electrolysis}:2{\mathrm{CO}}_{2}↔\mathrm{CO}+O_{2}$$

(14) $$\mathrm{Sabatier}\;\mathrm{reaction}:\;CO_{2}+4H_{2}↔CH_{4}+2H_{2}O$$

(15) $$\mathrm{Bosch}\;\mathrm{reactrion}:CO_{2}+H_{2}↔\mathrm{CO}+H_{2}O$$

(16) $$2\mathrm{CO}↔CO_{2}+C(s)$$

(17) $$\mathrm{CO}+H_{2}↔H_{2}O+C(s)$$

SOEC technology offers a direct method for oxygen production and serves as a key component in the carbon cycle for ISRU. It is considered one of the most promising and practical approaches for converting the Martian atmosphere—composed of ≈96% CO_2_—into usable oxygen, thereby reducing resource consumption. The Mars Oxygen ISRU Experiment (MOXIE), developed by the Massachusetts Institute of Technology (MIT) using SOEC technology, is a facility designed to produce oxygen from CO_2_ (Figure 11c).^[^ ^209^ ^]^ Currently, there are three primary requirements for MOXIE technology: First, the oxygen generation rate must be no less than 6 g hr^−1^, assuming an atmospheric inlet pressure of 5 Torr, typical of the rover's interior environment. Second, the purity of the produced oxygen must exceed 98%. Lastly, the system must be capable of operating for at least ten cycles after delivery while meeting the first two criteria. In the process of converting CO_2_ to O_2_, MOXIE employs ten solid‐state electrolysis cells for oxidative electrolysis, divided into two groups of five cells each. Under a current of 4 A, this arrangement of electrolyzers can achieve an oxygen production rate of 12 g hr^−1^.^[^ ^209^ ^]^

While SOEC‐based technologies like MOXIE are highly effective for oxygen extraction from CO_2_ in atmospheric environments such as Mars, alternative approaches are required for airless celestial bodies such as the Moon, where CO_2_ is not readily available. In this context, Zhong et al. developed an alternative lunar ISRU strategy based on electrocatalytic CO_2_ reduction using Chang'e‐5 (CE‐5) lunar soil samples. By loading copper onto lunar regolith, they significantly enhanced its catalytic activity, enabling high‐efficiency CO_2_‐to‐methane and oxygen conversion in a flow cell electrolyzer. Their results demonstrated that the Cu/MgSiO_3_ catalyst achieved a Faradaic efficiency of 72.05% for CH_4_ at a current density of 600 mA cm^2^, with an oxygen evolution rate of 2.3 mL min^−1^, maintaining high stability even at 800 mA cm^2^. Furthermore, the research team developed an automated robotic system that enables unmanned catalyst preparation and electrolysis operations, which is essential for long‐duration lunar missions. Ultimately, this study validated that lunar soil‐based CO_2_ reduction can serve as a sustainable pathway for methane and oxygen production, offering a hydrogen‐independent CO_2_ utilization approach that is highly suitable for the lunar environment. This breakthrough provides a critical technological foundation for energy self‐sufficiency in lunar bases and paves the way for sustainable deep‐space exploration.^[^ ^210^ ^]^

## 5 Energy Storage

**Figure 12:**
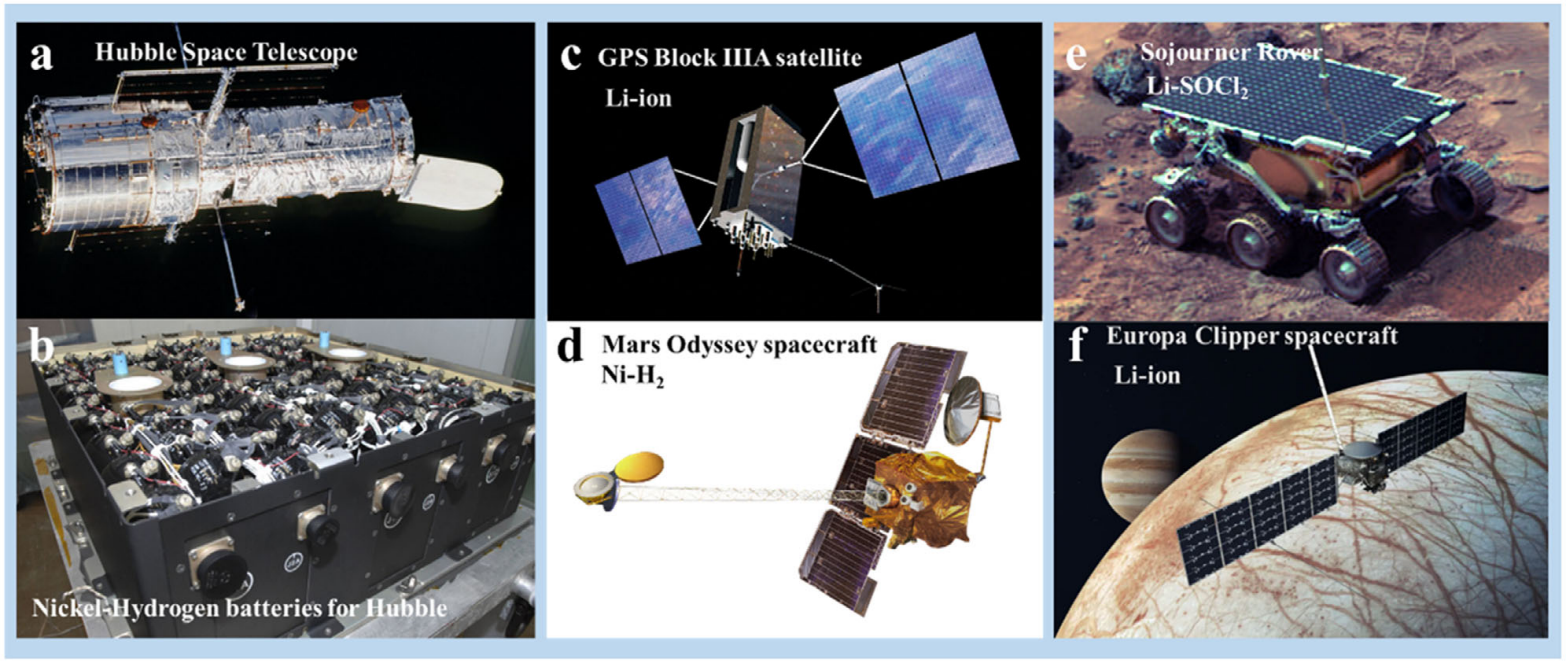
Composite illustration of spacecraft systems powered by advanced battery technologies. a) The Hubble Space Telescope operating in low Earth orbit. Image credit: NASA / Public domain. b) Nickel‐hydrogen battery module used onboard the Hubble mission. Image courtesy of NASA / Public domain. c) GPS Block IIIA satellite with power‐regulated solar and battery systems. Image credit: U.S. Air Force/Public domain. d) Mars Odyssey orbiter conducting planetary remote sensing. Image credit: NASA/JPL/Public domain. e) Sojourner rover on Mars, powered by solar‐charged batteries during the Pathfinder mission. Image courtesy of NASA/JPL. f) Europa Clipper spacecraft, developed for Jupiter system exploration, powered by solar arrays and radiation‐tolerant batteries. Image credit: NASA/JPL‐Caltech/Public domain.

In spacecraft launches and long‐term exploration, batteries have proven critical for addressing the timeliness and safety challenges of energy supply, including primary batteries in landers, thermal batteries in reentry capsules, and secondary batteries in rovers (**Figure** **12**a–f).^[^ ^213^, ^214^ ^]^ Meanwhile, the rapid depletion of fossil fuels and escalating environmental costs make reliance on non‐renewable energy sources increasingly untenable for future space endeavors, thereby heightening the need for clean energy. Solar energy, in particular, offers significant potential for efficient collection and application on the Moon and other celestial bodies.^[^ ^215^ ^]^ Research indicates that certain regions on the Moon receive nearly continuous sunlight throughout the year, providing a solid foundation for solar energy utilization.^[^ ^216^ ^]^ Nevertheless, the deployment of solar and wind energy remains constrained by factors such as intermittency and site dependence, while installing large‐scale wind turbines in low‐density atmospheres introduces further technical challenges.^[^ ^217^ ^]^ Consequently, energy storage systems—batteries in particular—serve as critical mechanisms for balancing supply and demand. Primary batteries provide high‐reliability, single‐use power for short‐term, mission‐critical operations, whereas secondary batteries offer rechargeable, extended power solutions. Together, they form an integral part of modern spacecraft, underpinning sustainable energy strategies for future space exploration.

### 5.1 Primary Batteries

Primary batteries are single‐use batteries that cannot be recharged once their chemical reactants are depleted. This category includes thermal batteries, which have been crucial in space applications due to their reliability and high‐power output. The initial adoption of primary batteries in space exploration was necessitated by the challenge of harnessing solar energy in environments with limited sunlight.^[^ ^218^ ^]^ Early examples include water‐based systems, such as the silver‐zinc batteries used in the 1957′s Sputnik mission,^[^ ^219^ ^]^ and lithium‐based batteries like Li‐SO_2_ and Li‐SOCl_2_, which have been utilized in more recent missions.^[^ ^218^ ^]^ Thermal batteries, in particular, play a vital role in supporting high‐power pulses, such as those required for firing pyrotechnic charges during critical spacecraft operations.^[^ ^220^ ^]^ Among lithium‐based systems, Li‐SO_2_ batteries are the most commonly employed in aerospace applications. These batteries belong to the liquid cathode category, where the catholyte—a 1 M solution of sulfur dioxide in acetonitrile with lithium bromide—functions as both the cathode and electrolyte. The anode consists of lithium metal. During discharge, sulfur dioxide is reduced on a carbon cathode current collector, forming insoluble lithium dithionate as the reaction product.

To optimize performance, these batteries often adopt spirally wound designs, which enhance specific energy, minimize voltage delays, improve low‐temperature functionality (down to −40 °C), extend shelf life (>10 years), and increase pulse power capabilities.^[^ ^218^ ^]^ Li‐SO_2_ batteries have been deployed in missions such as the 2011 Mars Science Mission. Beyond Mars exploration, NASA has utilized these batteries in the Galileo and Huygens probes, the Genesis and Stardust sample return capsules, and the Mars Exploration Rovers’ lander systems.^[^ ^218^, ^219^ ^]^

### 5.2 Secondary Batteries

**Figure 13:**
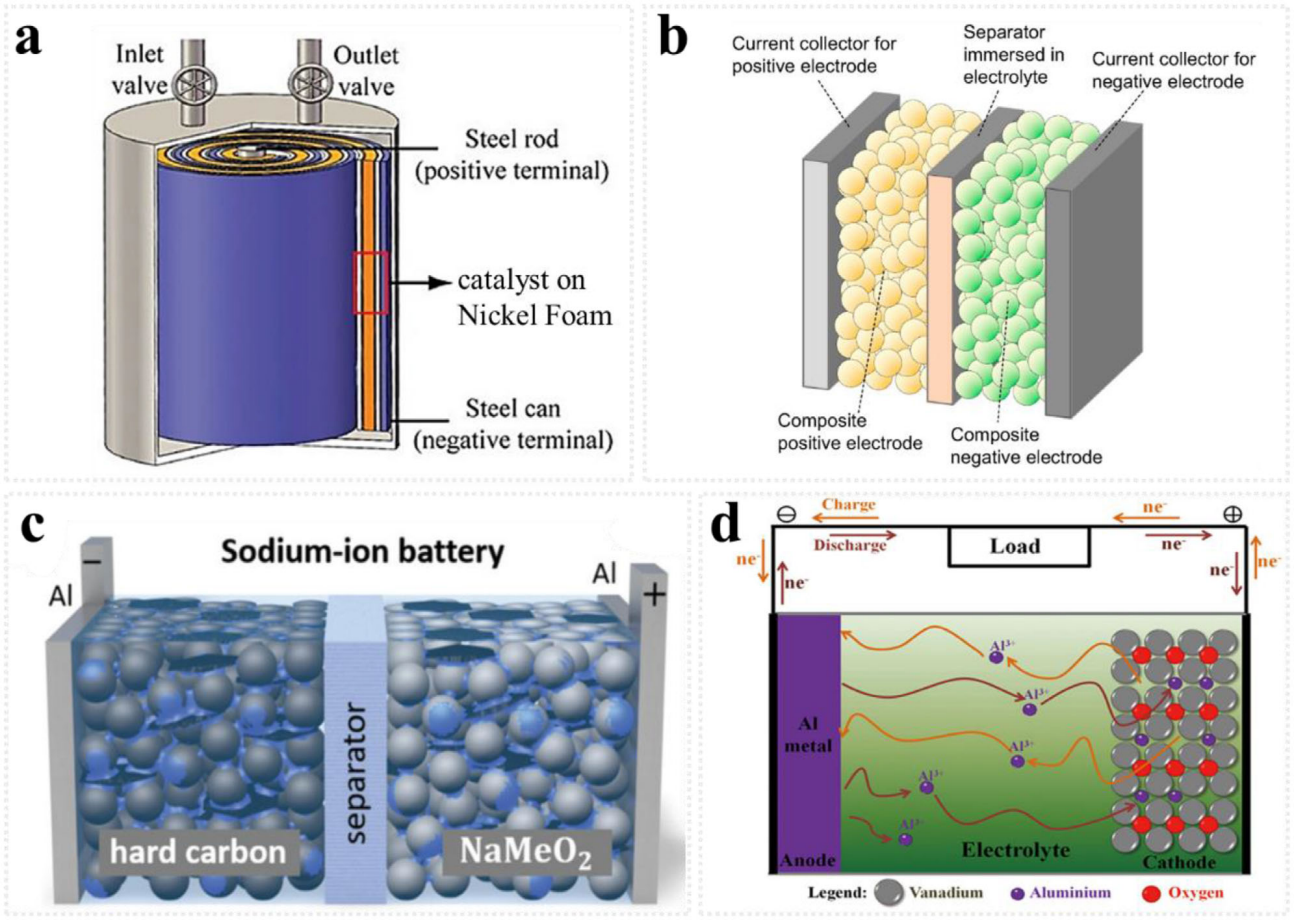
a) Schematic diagram of a nickel–hydrogen (Ni–H₂) battery. Reproduced with permission.^[^ ^225^ ^]^ Copyright 2024, Wiley‐VCH GmbH. b) Schematic diagram of a lithium‐ion battery (LIB). Reproduced with permission.^[^ ^242^ ^]^ Copyright 2017, Elsevier. c) Schematic diagram of a sodium‐ion battery. Reproduced under terms of the CC‐BY license.^[^ ^243^ ^]^ Copyright 2020, The Kudakwashe Chayamubenge et al., published by Wiley‐VCH GmbH. d) Schematic illustration of overpotential behavior during battery charging and discharging. Reproduced under terms of the CC‐BY license.^[^ ^244^ ^]^ Copyright 2013, The Wei Wang et al., published by Springer Nature.

Secondary batteries are rechargeable energy storage devices, designed for repeated cycles of charge and discharge. These batteries are categorized based on their electrode materials and electrolytes into several types, including lead‐acid (LA), nickel‐cadmium (Ni‐Cd), nickel‐hydrogen (Ni–H_2_) (**Figure** **13**a), lithium‐ion (Li‐ion) (Figure 13b), sodium‐ion (Na‐ion) (Figure 13c), and metal‐air (M‐A) batteries.^[^ ^221^ ^]^ Secondary batteries are indispensable for sustainable energy storage in long‐term space missions, enabling continuous power supply and adaptability to extreme environmental conditions.

LA batteries, among the earliest secondary battery systems, remain widely used due to their low cost, high unit energy, and good stability across varying temperature environments. They consist of a sponge lead anode, a lead dioxide cathode, and a sulfuric acid electrolyte. However, their limited cycle life, slow charging rates, high self‐discharge, and environmental hazards significantly restrict their long‐term suitability for space applications.^[^ ^222^ ^]^

Ni‐Cd batteries established themselves as a primary choice for early satellite missions, attributed to their exceptional low‐temperature performance and robust cycling stability. Furthermore, their high tolerance to overcharge and deep discharge rendered them particularly advantageous for applications in low Earth orbit (LEO) missions. Nevertheless, cadmium toxicity and moderate energy density have led to their gradual replacement by safer and higher‐performance alternatives.^[^ ^223^, ^224^ ^]^

Moreover, according to their electrochemical reaction equations, both LA and Ni‐Cd batteries generate gases at their electrodes during operation. Although explicit studies specifically addressing gas accumulation impacts under microgravity conditions for these batteries are lacking, considering the documented negative effects of gas bubble formation on electrode efficiency in water electrolyzers in microgravity, it is reasonable to infer that gas bubble accumulation has likely contributed to the diminished efficiency and operational lifespan of LA and Ni‐Cd batteries under microgravity conditions. This issue potentially serves as one contributing factor to their observed reduced popularity and gradual phase‐out in current aerospace applications.

Ni‐H_2_ batteries represent one of the most reliable battery systems ever deployed in space. Successfully used by NASA for decades in geosynchronous satellites and the International Space Station, they offer unparalleled longevity (often exceeding 100 000 cycles), robust performance in vacuum, and excellent thermal stability.^[^ ^225^ ^]^ Their core electrochemical mechanism is based on the reversible redox reactions between Ni(OH)_2_/NiOOH and H_2_/H^+^, with hydrogen stored in a pressurized vessel and oxygen evolved at the nickel electrode.^[^ ^225^, ^226^, ^227^ ^]^ These systems are particularly well‐suited for deep‐space and orbital applications where consistent power delivery and durability are essential.

Recent innovations have significantly enhanced Ni‐H_2_ technology. For example, Jiang et al. developed a highly active RuNi/C catalyst via ultrafast electrical pulse synthesis, achieving a HOR mass activity of 2.34 A mg^−1^ at 50 mV (versus RHE) and an HER overpotential of just 19.5 mV at 10 mA cm^−2^. This advancement enabled a Ni‐H_2_ battery to operate across −25 to + 50 °C with an energy density of 183 Wh kg^−1^ and stack‐level cost of only $49 kWh^−1^, making it suitable for both orbital missions and scalable terrestrial storage.^[^ ^227^, ^228^ ^]^

In addition, the development of pH‐universal, bifunctional catalysts such as PtRuNi aerogels has extended the electrolyte compatibility of Ni‐H_2_ batteries, enabling their application in diverse system designs.^[^ ^228^ ^]^ The synergy of high safety, ultra‐long life, low cost (via catalyst optimization), and robust temperature tolerance makes Ni‐H_2_ batteries a compelling choice not only for satellites but also for future lunar bases and space stations.

Lithium‐ion batteries have revolutionized space energy storage due to their high energy density and relatively low weight. First adopted in Mars rovers such as Opportunity and Spirit, Li‐ion cells have since become widespread in modern satellite and robotic systems. Conventional Li‐ion configurations employ graphite anodes and LiCoO_2_ or NMC as the cathode in organic electrolytes. These batteries became the primary energy source for Mars exploration missions, such as Opportunity and Spirit, due to their high energy density and long cycle life.^[^ ^229^ ^]^ However, safety concerns, such as thermal runaway during overcharging or short‐circuiting, remain a significant challenge. Additionally, the highly toxic and flammable organic solvents used in Li‐ion batteries raise environmental and operational risks.^[^ ^214^ ^]^ To address these issues, aqueous Li‐ion batteries were introduced as a safer and more cost‐effective alternative. These systems eliminate the need for organic solvents, thereby reducing flammability and simplifying manufacturing processes. For example, an early aqueous lithium‐ion battery employed VO_2_ as the anode and LiMn_2_O_4_ as the cathode, achieving a specific energy of 75 Wh kg^−1^ at ≈1.5 V.^[^ ^230^ ^]^ Despite their advantages, these batteries exhibit poor cycle life, with many systems retaining less than 50% capacity after 100 cycles (e.g., LiV_3_O_8_/LiNi_0.81_Co_0.19_O_2_ or TiP_2_O_7_/LiMn_2_O_4_).^[^ ^231^ ^]^

Recent advancements have significantly improved aqueous lithium‐ion battery performance. Luo et al. demonstrated that optimizing electrolyte pH, eliminating oxygen, and using carbon‐coated electrodes could enhance cycle life. Their LiTi_2_ (PO_4_)_3_/Li_2_SO_4_/LiFePO_4_ system retained over 90% capacity after 1000 cycles, even under rapid charge‐discharge conditions.^[^ ^232^ ^]^ Additionally, McDowell et al. explored lithium deposition and SEI layer evolution at extreme temperatures, demonstrating that lithium batteries could operate effectively at temperatures as low as −80 °C. This advancement overcomes the limitations of −20 °C operation, making lithium batteries suitable for extreme environments.^[^ ^233^ ^]^

Na‐ion batteries have gained attention due to the abundance and low cost of sodium compared to lithium. However, early sodium batteries suffered from low energy density due to sodium's large atomic radius and lower ionic conductivity.^[^ ^234^ ^]^ Innovations, such as using sodium alloys as anode materials and SnSb/C nanocomposites as cathodes, have greatly improved energy density and cycle life.^[^ ^235^, ^236^ ^]^ Multivalent metal‐ion batteries (Figure 13d), such as aluminum‐ion batteries, offer promising alternatives for energy storage. Aluminum‐ion batteries leverage aluminum's multivalent nature, allowing higher energy storage capacities. Their discharge and charge reactions are as follows:

Discharge reaction:

(18) $$\mathrm{Anode}:\mathrm{Al}-3e^{-}\to Al^{3+}$$

(19) $$\mathrm{Cathode}:VO_{2}+\mathrm{xA}l^{3+}+3xe^{-}\to Al_{x}VO_{2}$$

Charge Reaction:

(20) $$\mathrm{Anode}:Al^{3+}+3e^{-}\to \mathrm{Al}$$

(21) $$\mathrm{Cathode}:Al_{x}VO_{2}+\mathrm{xA}l^{3+}-3xe^{-}\to VO_{2}$$

During operation, aluminum ions migrate between the anode and cathode, with external circuits compensating for electron transfer. Aluminum‐ion batteries offer high energy densities, lower costs, and improved safety compared to Li‐ion systems. Wang et al. demonstrated through first‐principles simulations that aluminum‐ion batteries achieve an initial capacity of 165 mAh g^−1^, retaining 11 mAh g^−1^ after 100 cycles, indicating their high current density and potential for large‐scale applications.^[^ ^237^ ^]^

While traditional technologies like LA and Li‐ion batteries continue to serve crucial roles, advanced systems such as Ni‐H_2_ and Na‐ion are reshaping the landscape of space energy storage. Tailoring battery chemistry to mission‐specific demands is essential for enabling reliable, safe, and efficient power supply across increasingly ambitious exploration endeavors.

It is also noteworthy that some secondary battery technologies initially developed for space exploration have gradually evolved to benefit civilian applications. For example, although Ni‐H_2_ batteries were originally engineered for long‐term missions in geosynchronous satellites and the International Space Station due to their outstanding cycle life and environmental robustness, recent advancements have enabled their adaptation for terrestrial grid‐scale energy storage.^[^ ^238^, ^239^, ^240^, ^241^ ^]^ Through catalyst optimization and cost reduction strategies, Ni‐H_2_ systems have been successfully commercialized for stationary storage, offering high reliability and longevity suitable for renewable energy integration.^[^ ^241^ ^]^ This transition exemplifies how technologies born from the stringent demands of space missions can, in turn, contribute to advancing sustainable energy solutions on Earth.

## 6 Summary And Outlook

Electrochemical systems exhibit transformative potential in space exploration through molten salt electrolysis, solid‐state electrolyzers, and intelligent control architectures. Laboratory investigations have demonstrated that electrolysis of ilmenite (FeTiO_3_) in simulated lunar regolith can achieve oxygen yields exceeding 90%, directly supporting NASA's Artemis program for sustained lunar habitation.^[^ ^48^, ^245^, ^246^ ^]^ Furthermore, the Mars Oxygen In‐Situ Resource Utilization Experiment (MOXIE) aboard NASA's Perseverance rover has successfully generated oxygen from Martian atmospheric CO_2_ at a rate of 5.4 g h^−1^, validating the robustness of electrochemical systems under extraterrestrial conditions.^[^ ^247^ ^]^ While yttria‐stabilized zirconia (YSZ)‐based SOECs operate optimally at elevated temperatures (500–1000 °C), PEM systems demonstrate efficiency at lower temperatures (≈80 °C). However, both technologies must be adapted to extraterrestrial thermal extremes, including the Moon's 300 °C diurnal temperature fluctuations and Mars’ average surface temperature of −60 °C.^[^ ^248^, ^249^ ^]^

Despite these advances, extreme environmental conditions and resource constraints pose multifaceted challenges. In microgravity environments, gas bubble adhesion on electrode surfaces can reduce reaction efficiency by up to 40%.^[^ ^250^, ^251^ ^]^ However, the implementation of nanoporous nickel electrodes, as developed by JAXA, mitigates this issue by reducing bubble detachment size. Radiation resistance is another critical factor, with silicon carbide (SiC) coatings enhancing the durability of PEM systems and mitigating degradation effects.^[^ ^252^, ^253^ ^]^ Resource limitations necessitate innovative solutions, such as electrodialysis and microbial electrolysis cells (MECs), which have shown promise in terrestrial water treatment applications. However, further research is required to validate their efficiency in processing Martian ice deposits, as specific recovery rates under extraterrestrial conditions remain uncertain.^[^ ^254^ ^]^

Future advancements will require interdisciplinary innovation. Emerging materials such as high‐entropy alloy electrodes (e.g., FeCoNiCrMn) and self‐healing electrolytes (e.g., vitrimers) offer promising avenues for enhancing corrosion resistance and operational durability.^[^ ^255^ ^]^ At the system level, AI‐driven optimization—exemplified by MIT's machine learning framework, which reduces energy consumption in electrochemical processes—combined with digital twin technologies, will enhance adaptability to extreme extraterrestrial conditions. Autonomous maintenance solutions, such as NASA's Astrobee robotic system, can further support in‐situ repairs, while additive manufacturing holds potential for fabricating electrochemical infrastructure on‐site.^[^ ^256^ ^]^ Beyond space applications, terrestrial deployment of these advanced electrochemical technologies could accelerate global energy transitions and contribute to carbon neutrality initiatives.

In conclusion, electrochemical systems represent a cornerstone for sustainable off‐world habitation. However, their large‐scale implementation necessitates overcoming challenges related to environmental compatibility, closed‐loop resource utilization efficiency, and intelligent process control. (**Table** **1**) By integrating multiscale innovations spanning materials science, systems engineering, and planetary resource analytics—underpinned by international collaboration—humanity may realize the vision of self‐sustaining “electrochemical ecospheres”, paving the way for deep‐space exploration and interplanetary colonization. Meanwhile, technological breakthroughs achieved through space‐oriented electrochemical research are increasingly serving as a bridge between extraterrestrial exploration and terrestrial technological innovation. In particular, secondary battery technologies, initially developed to meet the stringent demands of space missions, have significantly advanced civilian energy storage solutions, fostering the growth of grid‐scale storage systems and electric mobility.^[^ ^225^ ^]^ In this way, space‐driven electrochemical advancements contribute both to sustaining human activities beyond Earth and to addressing critical sustainability challenges within our planetary boundaries.

**Table 1:** Summary of key differences between terrestrial and space environments for electrochemical technologies.

Research Area	Major effects in space environment	Advantages in terrestrial environment	Key challenges
**Molten Salt Electrolysis/** **Metallurgy**	Reduced gravity delays gas bubble detachment, lowering efficiency^[^ ^94^, ^126^ ^]^	Gravity facilitates bubble separation, resulting in higher efficiency^[^ ^94^, ^126^ ^]^	Electrode design optimization to reduce gas stagnation^[^ ^94^ ^]^
**Water Electrolysis**	Gas bubbles accumulate and block electrode surfaces, decreasing efficiency^[^ ^129^, ^131^, ^257^ ^]^	Easier bubble detachment, higher efficiency due to buoyancy^[^ ^129^, ^131^, ^257^ ^]^	Enhancement of bubble removal via artificial gravity methods^[^ ^126^ ^]^
**Electrochemical CO_2_ Capture and Conversion**	Microgravity influences gas‐liquid interactions, increasing overpotential^[^ ^258^ ^]^	More favorable terrestrial conditions, mature technological infrastructure^[^ ^258^ ^]^	Enhancement of bubble removal via artificial gravity methodsEnhancement of bubble removal via artificial gravity methods^[^ ^258^ ^]^
**Battery Energy Storage**	Microgravity complicates thermal management; radiation accelerates degradation^[^ ^218^, ^259^ ^]^	Natural convection cooling and lower radiation exposure^[^ ^218^, ^259^ ^]^	Thermal management optimization and radiation‐resistant materials development^[^ ^126^, ^218^ ^]^

## Conflict of Interest

The authors declare no conflict of interest.
